# Engineering of FK520 polyketide synthase for rapid access to quality control reference standards

**DOI:** 10.1186/s12934-025-02861-3

**Published:** 2025-11-28

**Authors:** Nina Žigart, Petra Pivk Lukančič, Tjaša Drčar, Jan Peterka, Maja Harej Perko, Peter Mrak

**Affiliations:** 1 Sandoz Technical Operations, MS&T Antiinfectives, Kolodvorska 27, SI-1234 Mengeš, Slovenia; 2 Sandoz Development Center Slovenia, Ljubljana, Slovenia

**Keywords:** PKS engineering, AT domain, FK506, FK520, Pimecrolimus, Ascomycin, Tacrolimus, Impurity, Related compounds, Chlorination, Isoleucine, Analogues, Valine

## Abstract

Despite the ability of mega synthases to construct complex structures with high precision, the main product is often accompanied by minor congeners because of relaxed selectivity of biosynthetic steps. In the case of polyketides, these may arise from promiscuity of acyltransferase domains while selecting the extender building blocks. This yields compounds which can differ only slightly from the main product and are therefore notoriously difficult to separate. For compounds in medicinal use, such congeners are considered impurities and must be precisely quantified in commercial production through use of reference standards. The immunosuppressants FK506, FK520, and its chlorinated derivate pimecrolimus, are well known for accumulation of minor congeners in the fermentation, which demands costly and lengthy purification with industrial HPLC to obtain material of sufficient purity. The relaxed selectivity of the AT domain in module 4 of FK506/520 PKS results in production of FK506, FK506D, FK520, and FK523 from the same system. Here, we investigate additional minor impurities, specifically the 11-, 17-, and 19-ethyl FK520 derivatives. Because the PKS can process these analogues to completion, it is reasonable to expect that strategic replacement of AT domains in modules 9, 6, and 5 (respectively), would enhance their production. Indeed, we show a direct, exclusive and high titer biosynthesis of these analogues through PKS engineering, replacing tedious and costly isolation by preparative HPLC and significantly improving access to these minor congeners. In addition, by using the AT4 as the donor domain, an interesting system is obtained, containing identical promiscuous AT domains at two different positions, and consequently distinct intra- and intermodular context. Media-dependent extender pool modulation toward methylmalonyl-CoA showed unexpected but consistent distribution of the four predicted analogues for each of the engineered systems. Despite the use of identical ATs, extender incorporation preference is different, depending on the modular context. It appears that the selectivity of the downstream AT4-containing module depends on the structural features of the incoming acyl chain; features installed by a module well upstream in the assembly line. Therefore, the global context of the PKS may be more impactful to the outcome of AT-engineering experiments than is generally considered.

**Graphical abstract:**

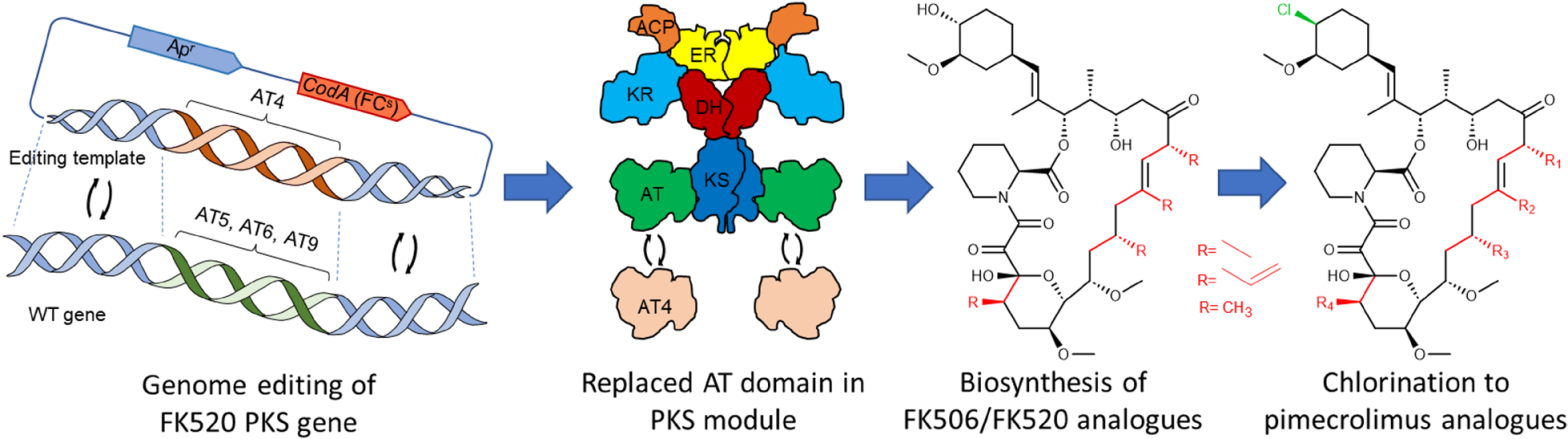

**Supplementary Information:**

The online version contains supplementary material available at 10.1186/s12934-025-02861-3.

## Background

Fermentative production of natural products is more often than not accompanied by accumulation of minor biosynthetic congeners. With “assembly-line” megazymes such as nonribosomal peptide synthetases (NRPS) and polyketide synthases (PKS), one reason for the accumulation of structural analogues lies in promiscuous behavior in starter/extender selection steps during the build-up of the molecular core. In the case of polyketides, this may occur due to the relaxed substrate specificity of acyltransferase (AT) domains (Table 1). These congeners pose significant challenges in industrial purification processes and consequently critically impact the cost of the product. In addition, the related compounds must be precisely quantified in pharmaceutical quality control, which typically demands access to gram quantities of pure compounds to serve as reference standards [1]. For the minor congeners accumulating in trace amounts in the fermentation culture, this is challenging and demands tedious and costly purification from industrial waste fractions in low yields, often requiring several successive preparative HPLC steps. Synthetic access to these compounds is even more impractical as total synthesis is notoriously difficult, with low yields and itself burdened by the generation of additional side products [2].

**Table 1:** Selected examples of polyketides in medicinal use and their congeners originating from relaxed selectivity of PKS AT domains

Compound	Analogue	Misincorporation^a^	Ref.
monensin A	monensin B monensin C	AT5 emal >mmal AT5 emal >mmal	[3]
geldanamycin	6-methyl	AT5 moxmal >mmal	[4]
epothylone A	epothylone B	AT3 mal >mmal	[5]
avermectin	24-desmethyl	AT1 mmal >mal	[6]
FK520 (**1**)^b^	FK523 (**4**) 11-ethyl (**5**) 11-ethyl (**6**) 19-ethyl (**7**)	AT4 emal >mmal AT9 mmal >emal AT6 mmal >emal AT5 mmal >emal	[7, 8], this work
rapamycin	11-ethyl 23-ethyl 31-ethyl	AT13 mmal >emal AT7 mmal >emal AT3 mmal >emal	[9, 10]
FK506 (**2**)	FK520 (**1**) FK506D (**3**) FK523 (**4**)	AT4 allmal >emal AT4 allmal >pmal AT4 allmal >mmal	[11, 12]

^a^ The predominant extender is listed first, followed by the misincorporated extender of the congener. Extenders: mal, malonyl-CoA; emal, ethylmalonyl-CoA; mmal, mehtylmalonyl-CoA; allmal, alylmalonyl-CoA; moxmal, methoxymalonyl-CoA; pmal, propylmalonyl-CoA. ^b^ The structural analogues are carried over in chlorinated form to pimecrolimus

**Fig. 1:**
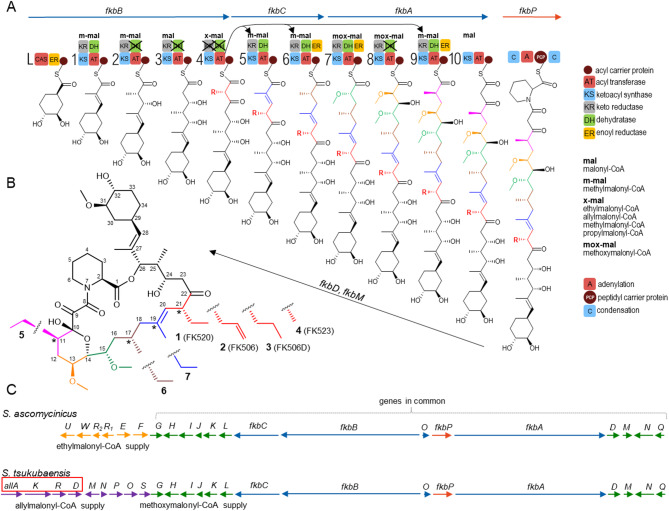
Biosynthesis of FK520 and FK506 in *S. ascomycinicus* and *S. tsukubaensis*, respectively. **A** PKS assembly line with extender unit utilization and polyketide condensation sequence. Each module is numbered and depicted with the encoded catalytic domains. The inactive domains are crossed out. Black arrows mark the positions of the donor AT4 and the domains swapped in this work. **B** Structure of FK520, FK506 and their congeners. * Marks the positions of naturally occurring ethyl congeners which are the focus of this work. **C** Genetic organization of *S. ascomycinicus* FK520 and *S. tsukubaensis* FK506 BGCs. The boxed genes (*allA*, *K*, *R* and *D*) in the FK506 BGC were used to introduce allmal supply to *S. ascomycinicus* (Fig. S11)

A well-known case in this respect, and the focus of our work, are the immunosuppressants FK520 (**1**) and FK506 (**2**). These medically important polyketide macrolactams, produced by *Streptomyces ascomycinicus* and *Streptomyces tsukubaensis*, respectively, are built up by a modular type I PKS and completed with a NRPS step, enclosing the nascent chain into a macrolactame (Fig. 1 [13, 14]). Modular type I PKS act as assembly lines, sequentially incorporating 2-substituted malonyl-CoA building blocks into the growing chain. Each module is composed of core catalytic domains: β-ketosynthase (KS), acyltransferase (AT), and acyl carrier protein (ACP), optionally amended by reductive loop domains: ketoreductase (KR), dehydratase (DH), and enoyl reductase (ER). Based on domain composition, the reductive loop reduces the newly formed β-keto group to a certain degree [15]. Although the 11-module PKS of the FK506 and FK520 biosynthetic pathways are essentially the same, the resulting major products differ in the side chains at carbon 21. This difference originates primarily from distinct extender-CoA supply genes in the biosynthetic gene clusters (BGC). While the FK506 pathway of *S. tsukubaensis* contains the “*All* subcluster”, encoding allylmalonyl-CoA (allmal) synthesis, the FK520 biosynthetic gene cluster (BGC) of *S. ascomycinicus* harbors the ethylmalonyl-CoA (emal) genes (Fig. 1 [11, 12]). In addition, there is some difference in the extent of the relaxed specificity of the AT domain in module 4 between the FK506 and FK520 PKS [16].

Due to the dual role of the allmal pathway supplying both allmal and emal to the cellular pool [17], *S. tsukubaensis* coproduces both FK506 and FK520 from the same PKS. Furthermore, a propylmalonyl-CoA (pmal) misincorporation product FK506D (**3**), can be found in cultures of *S. tsukubaensis* [11, 12]. The *S. ascomycinicus* pathway also displays diversification of products at C21; in addition to FK520, its desmethyl analogue FK523 (**4**) can be coproduced in significant quantity [8, 18], suggesting incorporation of methylmalonyl-CoA (mmal) instead of emal by module 4 of the PKS. All the above indicates that the AT domain of module 4 is highly promiscuous in loading the extender-CoAs. As the ultimate proof, experiments with the external supply of SNAC-tethered extenders in the absence of their native cluster-encoded biosynthesis showed that it is possible to direct the outcome of the pathway exclusively toward either FK520 or FK506 [19]. Additionally, through metabolic engineering and media optimization, it has been shown that the rate of incorporation of a certain extender-CoA moiety by such promiscuous ATs largely depends on the relative availability of the competing substrates from the cellular pool [12, 18, 20, 21]. To this end, our study shows that by simple modification of the growth medium, one can drastically change the outcome of the FK520 biosynthetic pathway.

It is not well known that *S. ascomycinicus* accumulates several other minor structural variants of FK520, which likely originate from AT domain promiscuity by misincorporation of emal instead of mmal. These are the 11-ethyl (**5**), 17-ethyl (**6**), and 19-ethyl (**7**) analogues of FK520 [7, 8]. Together with the structural diversity found at C21, this indicates that the FK520 biosynthetic machinery, in general, is capable of successful completion of structural analogues at various positions, i.e. the structural changes in the growing polyketide chain do not critically hinder the downstream biosynthetic steps. Exploiting this fact and following up on previous PKS engineering work [20, 22], we have set to explore whether the introduction of promiscuous AT domains such as AT4 elsewhere in the FK520 PKS allows further diversification of the pathway outcomes in a practical manner. By utilizing this promiscuity, one should be able to obtain multiple structural variants from each PKS-engineered strain through modulation of the cellular extender-CoA pool.

Therefore, modules 5, 6, and 9 were targeted for AT4 domain introduction, partly to obtain proof of concept, but also with a specific application in mind: the production of congeners **5**, **6**, and **7** individually and in significant amounts in order to drastically improve access to reference standards used in pharmaceutical quality control analytics for FK520 and its C32-chlorinated derivative pimecrolimus. An industrial FK520 producer, *S. ascomycinicus* H076, was selected for its high productivity as a chassis for this study.

## Results and discussion

### Modulation of pathway outcome by influencing the extender-CoA supply

**Fig. 2:**
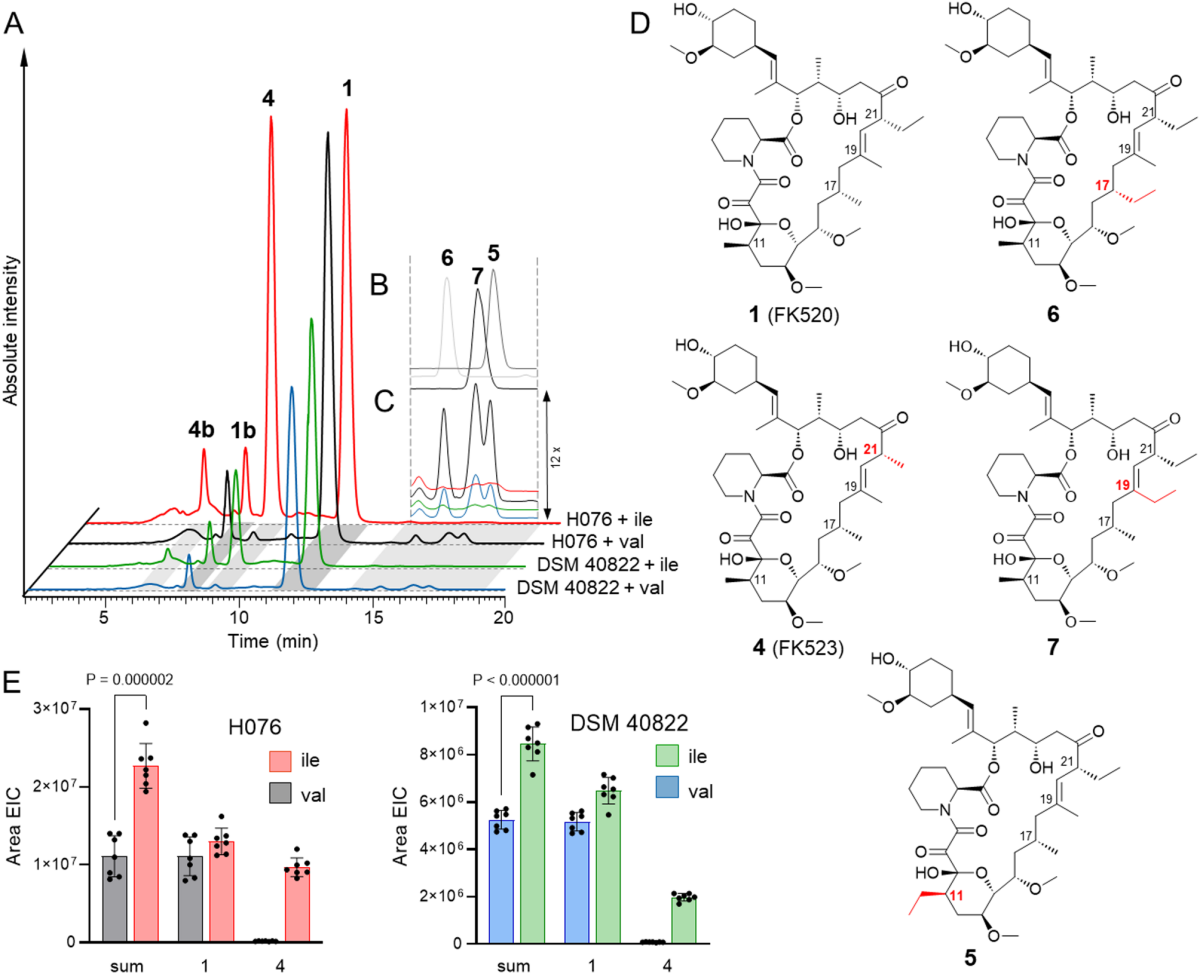
Accumulation of structural analogues of FK520 in cultures of *S. ascomycinicus* H076 and DSM 40,822 (ATCC 14891). **A** Extracted LC-MS chromatograms (*m/z* [M + NH_3_]^+^ = 795, 809 and 823) on an absolute intensity scale. **1b** and **4b** are isomers (epimerization at C10 via ring opening) in equilibrium with their respective main forms [23, 24]. **B** chromatogram overlay of isolated **5**, **6** and **7**, compared to **C** 12x zoom into chromatogram area containing ethyl analogues of FK520 (**5**, **6** and **7**). **D** Structures of FK520 and its analogues. **E** Cumulative accumulation of FK520 and **4** in cultures supplemented with Val and Ile (t test, *n* = 7)

LC-MS analysis of 5 day-old fermentation cultures of both *S. ascomycinicus* DSM 40,822 (ATCC 14891) and its super-producer derivative H076 (Fig. 2, Fig. S1) showed accumulation of several minor congeners in addition to the main product - the FK520 and its equilibrium isomer **1b** [23, 24]. The 21-desmethyl-FK520 analogue FK523 (**4**) [8], progenitor of the 21-desmethyl pimecrolimus impurity (**8**), was found at ~ 2% relative to FK520 with both strains. In addition, three compounds with *m/z* [M + NH_4_]^+^ = 823 (**5**, **6**, and **7**) were found accumulating in trace amounts in the cultures. **7** was reported, and others suspected before [8]. Their MS spectra and chromatographic behavior indicated that these are likely the progenitors of 11-ethyl (**9**), 17-ethyl (**10**), and 19-ethyl (**11**) impurities found in pimecrolimus [7]. If so, these compounds are formed by misincorporation of emal instead of mmal by PKS modules 5, 6, and 9. As reported previously [19], the relaxed specificity of certain AT domains can be exploited by modulation of the availability of extender units in the cellular pool. Similarly to our approach with *S. tsukubaensis* [20], we tested whether we could modulate the cellular availability of emal vs. mmal by modifying the culturing conditions. Indeed, by supplementing isoleucine (Ile, 12 g L^− 1^) to *S. ascomycinicus* cultures, we observed drastic changes in the ratios between **4** and FK520 in favor of the former (Fig. 2). In addition, the emal misincorporation congeners **5**, **6** and **7** could no longer be detected in the cultures (Fig. 2). This indicates that the ratio between the corresponding module 4 extenders shifted strongly in favor of the mmal pool. Conversely, the addition of valine (Val) or leucine (Leu) instead of Ile likely shifted this ratio in the direction of emal, as indicated by the decreased accumulation of **4** and increased levels of **5**, **6** and **7** relative to the FK520 (Fig. 2). Experiments showed a very clear dose-dependent effect in both directions.

Interestingly, the addition of Ile significantly increased the titers of FK520 in both *S. ascomycinicus* H076 and DSM 4082 (18% and 25%, respectively). The cumulative titer of polyketide products, compared to cultures with Val, increased even more (105% and 61%, respectively) (Fig. 2E). This indicates that in typical growth conditions prioritizing FK520 production [8, 18, 25], the productivity of the PKS is limited by extender unit supply (specifically of mmal), rather than the activity of the PKS machinery. While emal to mmal ratio and availability is influencing one module (M4), mmal is utilized by additional 5 modules (M1, M2, M5, M6 and M9; Fig. 1A). Stoichiometrically, the increase in PKS output therefore demands significantly higher mmal flux increase compared to emal. This huge limitation (and potential), which is rarely observed with industrial super producers, is a very inviting target for further FK520 industrial strain improvement work. The cumulative polyketide productivity observed in the presence of Ile could be redirected to FK520 by metabolic engineering to increase emal supply [25] while maintaining the optimal ratios between emal and mmal pools to control the accumulation of **4**. Engineering of module 4 to gain higher emal selectivity (e.g. by modifying AT domain) [16] would likely provide such control and increase productivity in conditions of increased mmal pool even without engineering of the primary metabolism.

The reason for the contrasting impact of Val and Ile on mmal and emal pools is not immediately apparent. While the catabolic pathway of Ile to propionyl-CoA in bacteria [26] has long been established and could indeed contribute to the mmal pool through carboxylation with propionyl-CoA carboxylase (PCC), a similar metabolic fate is often attributed to Val through the hydroxyisobutyryl-CoA pathway [27] (Fig. S3). Therefore, the effect we have observed cannot be explained through these connections. Yet, the same behavior was reported before [8] and recently also observed with *Streptomyces albus* by Gläser *et al*. [28], who propose that the reason could be both indirect (through regulatory changes to metabolism, induced by Val) as well as due to thus far underrated metabolic connection between Val and emal. Indeed, several studies done with *Streptomyces* hint at the possibility of such a connection through the catabolic product of Val, isobutyryl-CoA, and its isomerization to n-butyryl-CoA (isobutyryl-CoA mutase) [27–29] (Fig. S3). To yield emal, the latter would have to be directly 3-carboxylated, and indeed, the promiscuous acetyl/propionyl-CoA carboxylase from *S. coelicolor* was shown *in vitro* to carboxylate butyryl-CoA almost indiscriminately to acetyl/propionyl-CoA [30]. The effects of feeding propanol/butanol to *S. ascomycinicus* in a recent preprint [31] also fit well with this hypothesis as well as our results.

To get a glimpse of the metabolic changes, we performed a snap-shot differential analysis of intracellular acyl-CoA levels between Val and Ile-supplemented cultures of *S. ascomycinicus* H076 on day 3 of the cultivation, when the polyketide accumulation rate is the highest. While propionyl-CoA could not be reliably detected in either case, significantly higher levels of emal and butyryl/isobutyryl-CoA were measured in cultures supplemented with Val. Although low, slightly higher levels of mmal were found in the cultures with Ile, while butyryl/isobutyryl-CoA could be hardly detected (Fig. S4). These results are well in line with the above hypothesis. When considering mmal and emal stoichiometry in the product synthesis, and the productivity differences between the two culturing conditions, a possibility of mmal being the rate limiting factor in the cumulative productivity of the PKS is indicated. Further metabolic flux analysis is needed to fully confirm this.

### Introduction of AT4 domain into modules 5, 6 and 9

Although in low quantities, the native FK520 PKS is obviously capable of processing the structural analogues **5**, **6** and **7** to completion. Therefore, it is reasonable to expect that a strategic replacement of AT domains individually in PKS modules 5, 6, and 9 (M5, M6, and M9) with an emal-selective one, would result in mostly exclusive accumulation of the corresponding analogue to replace the native FK520 production in each of the engineered strains. Despite the AT4 domain of the FK520 PKS being highly promiscuous, the cultivation conditions with valine supplementation have shown good control over the emal vs. mmal cellular availabitily, resulting in predominant incorporation of emal by the FK520 AT4 (Fig. 2). For this reason and with a prospect of subsequently using the promiscuity of AT4 for further structural diversification, we decided on FK520 AT4 as the donor domain.

**Fig. 3:**
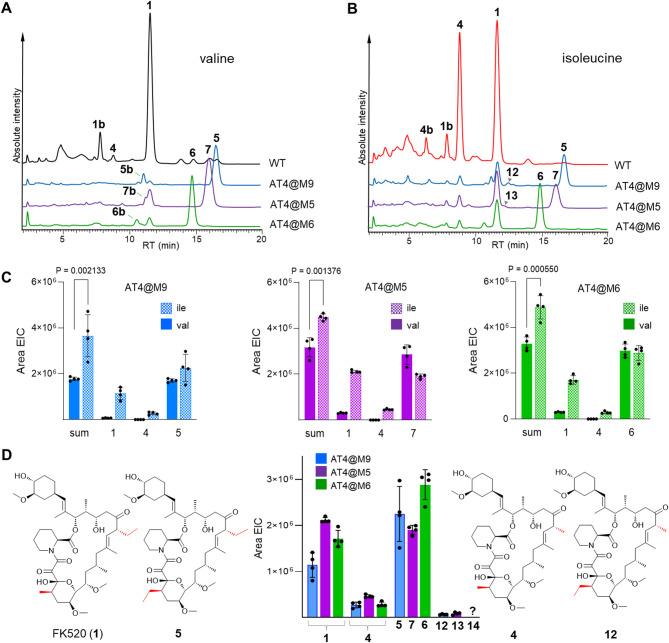
Accumulation of structural analogues of FK520 in cultures of AT replacement mutants in modules 5, 6, and 9 on the *S. ascomycinicus* H076 background. **A** LC-MS (ESI+) chromatograms (*m/z* = 700–900) of cultures supplemented with valine (12 g L^− 1^) on an absolute intensity vertical scale. **B** LC-MS (ESI+) chromatograms (*m/z* = 700–900) of cultures supplemented with isoleucine (12 g L^− 1^) on an absolute intensity vertical scale. **C** Accumulation of FK520 and analogues in cultures supplemented with Val and Ile (t test, *n* = 4). **D** Distribution of the four FK520 analogues found in cultures supplemented with isoleucine (12 g L^− 1^) and their structures on the example of AT4@M9

The domain exchange in the FK520 PKS was achieved with genome editing using a double crossover, positive-negative selection marker approach [32], Fig. S5). In all targeted modules, the exchange was performed in the consensus motifs at AT domain borders, leaving the upstream KS-AT and downstream AT-DH interdomain regions native to the targeted module untouched (Fig. S6). The genetic modifications were confirmed by PCR (Table S2, Fig. S7). The strains AT4@M5, AT4@M6 and AT4@M9 were then cultivated in shake flask cultures and analyzed for accumulation of FK520-related compounds with LC-MS. The results show that in all the engineered strains, the accumulation of FK520 practically disappeared. Instead, each of the strains accumulated a new major product with MS spectra (*m/z* [M + NH_4_]^+^ = 823) matching the expected incorporation of an additional emal instead of mmal (Fig. 3A, Fig S8). The structural analogues were accumulating in excellent titer (240–440 mg L^− 1^) and their retention times closely matched those of compounds found in traces in the WT cultures (Fig. 3A). These results conclusively confirm the biosynthetic origin of the “ethyl” FK520 congeners **5**, **6**, and **7**.

### Engineered PKS display reduced turnover

The productivity of AT replacement mutants was found to be significantly higher compared to similar AT domain replacement studies [20, 33–35]. Although the titers allowed access to gram quantities of the desired FK520 analogues in lab scale fermentation volumes, they were still only between ~ 15 and 27% of FK520 titer observed with the WT industrial super producer H076 (1603 ± 359 mg L^− 1^). To get more insight into where the bottlenecks may be, cultivation of mutants was also tested in the presence of Ile, thus allowing the engineered PKS and post-PKS machinery to run at least partly in overall native substrate mode through increased availability of mmal. Indeed, the changes in the mmal and emal pools, combined with the promiscuous nature of AT4 in both the original and the engineered modules, were directly reflected in accumulation of FK520 and **4** in addition to **5**, **6** or **7** (Fig. 3B) in the respective mutants. Although the combined productivity of Ile-supplemented cultures increased significantly again (compared to cultures with Val), the cumulative levels of PKS products and specifically the titers of native products FK520 and **4**, were significantly below those observed with WT strain under the same conditions (Figs. 2D and 3C). This indicates a reduced turnover of the engineered PKS.

AT domain engineering of assembly line PKS modules often disrupts complex and dynamic structural interdomain (and intermodular) interactions during the transacylation, elongation and reduction cycle, resulting in reduced processing rate or even inactivity [33, 36, 37]. With AT-swap experiments, it was found that this interdomain communication is not only affected by introduction of noncognate domains, but also by the changes introduced into the KS-AT-ACP interdomain regions (KAL and PAL), which were found to importantly influence the overall stability and activity of engineered PKS modules [38, 39]. No optimization of AT-flanking linker regions was performed in the chimeric modules in our case (KAL and PAL were left native in the receiving modules in our designs, Fig. S6). Although the AT-flanking interdomain regions are almost identical between modules 5, 6, and 9 of the FK520 PKS, there are considerable differences with module 4 (Fig. S6). The reduced productivity we have observed with the engineered PKS can be, at least in part, contributed to this fact.

Recent studies have also established a gatekeeping function of KS domains against noncognate incoming ketide chain substrates [40, 41]. This gatekeeping can cause stalling in the modules downstream of the AT exchange positions [41]. In our case, because of concurrent processing of cognate and of non-native substrates, KS domains downstream of the engineered modules (KS6 in strain AT4@M5, KS7 in strain AT4@M6 and KS10 in AT4@M9) may act in this manner, which would also contribute to reduced productivity of the system.

### Engineered PKS display peculiar product distribution

But there is another aspect to consider. Because each of the mutant PKS contains the promiscuous AT4 domain at two positions, one would expect accumulation of four distinct products under the increased mmal pool conditions in cultures supplemented by Ile. For example, AT4@M9 strain should produce **1** (21-ethyl, 11-methyl), **4** (21-methyl, 11-methyl), **5** (21-ethyl, 11-ethyl) and a fourth, novel analogue with the same MW as **1** (*m/z* [M + NH_4_]^+^ = 809) but with 21-methyl, 11-ethyl side chain combination (**12**, Fig. 3D). Based on results with WT H076 (AT4@M4) under the same conditions (Fig. 2A), a roughly equal distribution of these products was expected (with slight bias toward emal). Surprisingly, this was not the case, and the fourth analogue could not be found with any of the mutants until optimization of chromatographic method revealed a tiny peak next to **1** in AT4@M9 cultures (Fig. 3B, Fig. S8). Due to low concentration, the compounds could not be isolated to afford full structural confirmation, however the MS-MS fragmentation strongly supports the hypothesis that this is indeed the missing fourth analogue (Fig. S9). Similar observation was made with AT4@M5 (Fig. S8, Fig. S10). Unfortunately with the AT4@M6 mutant, judging from elution patterns, the fourth analogue could not be separated from **1**, so the data for this mutant could not be collected. Although the distribution data for the four analogues in AT4@M9 and AT4@M5 mutants show overall bias toward emal integration (likely coming from the extender pool ratios), they also strongly suggest different preference in integration of mmal vs. emal extenders of the modules containing otherwise identical AT4 domains (Fig. 3D, Info S1). Not only is the preference different but also seems to be interdependent; most notably, the engineered modules M9 and M5 are ~ 6 times and ~ 5 times (respectively) more likely to incorporate emal if emal was installed by the native module M4. Vice versa, the engineered modules strongly prefer mmal if methyl side chain is installed at C21 (Info S1). Interestingly, the distribution of products is similar with both AT4@M9 and AT4@M5 despite the significantly different positioning of structural variances in the polyketide chain.

While differences in selectivity of modules containing identical promiscuous AT domain, could be explained by different structural context within the module or by the gatekeeping function of downstream KS [40–43], it is more difficult to explain the observed interdependence in the product distribution. The current understanding of mechanisms governing intermodular communication [36, 37] is insufficient in this sense, mainly because with AT4@M9 these processes are occurring five modules apart. It is therefore reasonable to consider the possibility that structural differences in the incoming acyl chain (ethyl vs. methyl at C21) are driving the phenomenon. It has been recently shown that different KS (depending on the properties of the substrate tunnel) distinguish between various structural differences in the incoming ketide chain at least on the α, β and γ carbons, which results in different turnover rates [40]. It is plausible that this could apply also for significantly more distal structural variances on the substrate (11 carbons from the carbonyl group for the AT4@M9 substrate). Increasing body of evidence is emerging on the related fatty acid synthases, where KS condensation rates depend on the incoming acyl chain length and branching [44, 45]. With synthesis of branched chain fatty acids, relative rates of condensation with mal vs. mmal extenders are suspected to be influenced by the KS gatekeeping effects [45]. It is difficult to speculate on mechanistic reasons for our observations at this time, because in principle, ketide chain-dependent changes in kinetics of any of the catalytic functions within the engineered promiscuous module would change its product profile and could (through gatekeeping of a downstream KS) also result in changes in apparent extender preference. What can be concluded at this time is that our results show that restrictions in productivity and substrate preference of AT-swapped PKS are multifaceted, and in some cases likely unpredictable with the current understanding and tools.

### Purification, chlorination and comparison with classic approaches

**Fig. 4:**
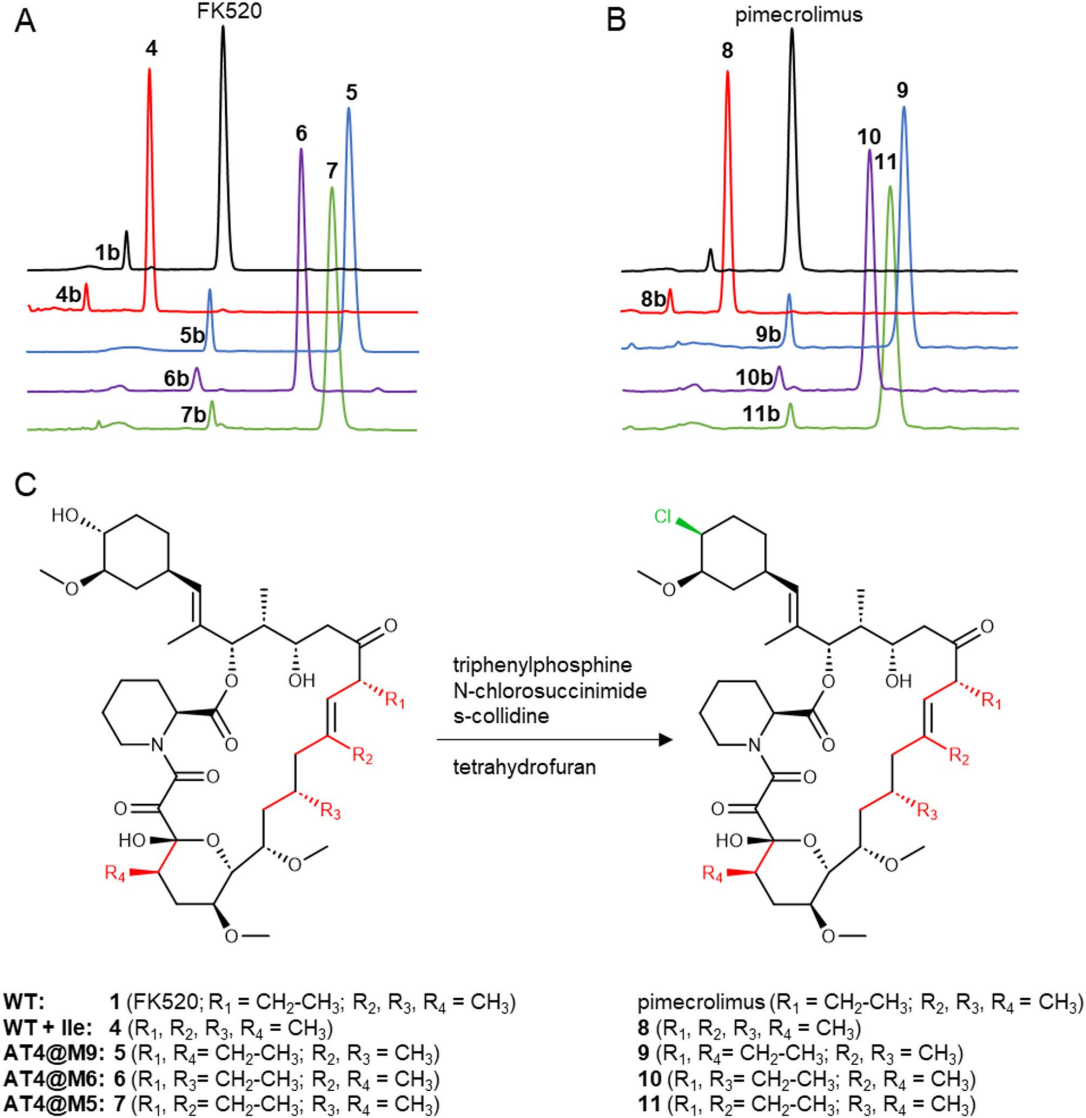
A single step, small scale chlorination of FK520 and its analogues to the corresponding pimecrolimus compounds. **A** Normalized UV chromatograms (λ = 210 nm) for isolated FK520 analogues and **B** their corresponding pimecrolimus derivatives after chlorination work-up. Note the presence of the equilibrium isomers in front of each main compound peak. **C** Synthetic scheme of the chlorination; a variation of Appel reaction. N-chlorosuccinimide (NCS) is the donor of chlorine for halogenation of TPP. This is followed by the formation of the alkoxide from the C32 hydroxyl group. Alkoxide subsequently attacks the phosphorous, releasing the halide leaving group. In a nucleophilic substitution reaction (S*N*2), the halide (nucleophile) attacks the C32, resulting in the final alkyl halide product with inverted stereochemistry

The fermentation was linearly scaled up from shake flask cultures to 2 L benchtop fermentors (1.6 L culture volume) using the same media and culture conditions. H076 supplemented with Ile (12 g L^− 1^) was used to obtain **4**, AT4@M5 mutant strain to obtain **7**, AT4@M6 strain to obtain **6** and AT4@M9 strain to obtain **5** (the latter three supplemented with 12 g L^− 1^ Val). Several parallel fermenters were dedicated to each strain. Titers after 160–210 h of fermentation were 280–420 mg L^− 1^ and extraction from each of the culture broths yielded between 400 and 700 mg (assay) of crude **4**, **5**,** 6** or **7** per fermenter. A single, high-load C18 prep-HPLC chromatography yielded compounds in high purity (86–94% area @ λ = 210 nm) in the form of white crystals, reminiscent of FK520. The structure was confirmed with ^1^H and ^13^C NMR (Info S2). A sub-gram scale, single-step chlorination protocol (Fig. 4) was developed based on [7] to prepare pimecrolimus analogues of **4**, **5**, **6**, and **7**. Reaction mixtures were analyzed with LC-MS, which confirmed chlorination yielding **8**, **9**, **10**, and **11**, respectively (Fig. 4, Fig. S12). In our initial attempts, the reaction was significantly hindered by reagent quenching (presumably due to contamination with water through solvents/reagents/atmosphere) and high evaporative loss during addition and sampling operations. Abandoning the classic, stirred, small volume reflux system in nitrogen atmosphere for simple sealed reaction tubes, incubated on a rotary shaker, resulted in a significant increase in conversion and yields of the chlorination. Good, 70–93% conversion was observed for all compounds, wherein a small part of the substrates remained unreacted and some of the chlorination products further degraded to their dehydrated product (23–24 dehydration, Table S6). A single, high-load prep-HPLC run afforded essentially pure **8**, **9**, **10**, and **11** after the solid-phase extraction of collected fractions (XAD1600, MTBE). The compounds were found identical to the authentic reference standards based on LC-MS analysis.

In our previous workflow, **8**, **9**, **10**, and **11** were isolated from industrial waste fractions in production of pimecrolimus where they are found in low concentrations. This demanded literally hundreds of preparative HPLC runs to collect gram quantities of material in pure form. The reason lies in almost identical chromatographic behavior of these and other structural analogues found in industrial processes (similar physical and chemical properties in general). In fact, **9** and **11** could not be chromatographically separated, even with high-resolution prep-HPLC setup. In contrast, our new approach turned out to be very straightforward and incredibly efficient. By providing the starting material (mutant strain fermentation broth) which contains only one of these congeners each, and at a good titer, the chromatography is no longer the principal separation method, but rather a polishing step removing mostly nonrelated compounds. Efficient, single step protocol for small scale chlorination further contributes to the efficiency of the approach.

## Conclusion

Engineering of modular PKS, particularly the AT domain swaps, is becoming a routine approach for obtaining structural analogues of natural products. Indeed, combining our study with previous engineering work on the FK506/FK520 PKS, various AT domain replacements have now been successful in most modules building up the calcineurin-binding side of the molecule (M4-M9) [20, 22]. In this work, we show that even with a very rudimental engineering design, gram quantities of artificial and minor natural analogues are within reach of research laboratories when performed on industrial super-producer strain chassis, with no need for semi-industrial equipment or laborious collecting of low-titer material. While PKS engineering techniques used to be reserved for basic research and drug discovery, we demonstrate their application for very practical tasks such as preparation of reference material for pharmaceutical QC analytics.

While the same outcome could be achieved by selecting a more emal-specific donor AT domain [16], our approach with the promiscuous AT4 domain from the FK520 PKS allowed additional insights into the likely bottlenecks in the productivity of both WT and engineered PKS strains. Our findings indicate that the apparent extender preference of a module containing a promiscuous AT domain may be influenced by structural differences in the incoming acyl-chain substrate which are much more distant to the reactive group than shown to date. While the mechanistic reasons for our observations are outside of the scope of this study, it is nevertheless clear from our results that the factors influencing the targeted engineering of the polyketides are not understood well enough to allow a reliable prediction of the outcome without empirical tests. Existence of even trace amounts of naturally present congeners can therefore be a valuable predictor for success of PKS engineering.

In this context, the use of promiscuous AT domains at various positions allows relatively simple feasibility studies on incorporation of more exotic 2-substituted malonyl-CoA extenders into the polyketide structure [46]. This requires the introduction of biosynthetic genes for the desired extenders or, alternatively, external supply of their synthetic thioesters such as SNACs [12, 19]. For illustration, we tested how the system behaves when allmal biosynthetic genes (*AllAKRD*) from *S. tsukubaensis* [11] are introduced to *S. ascomycinicus* strains. While the single AT4 domain at its native position in the WT strain afforded accumulation of ~ 5% of FK506 in addition to FK520 (which to our knowledge is the first report of engineered FK506 production in *S. ascomycinicus*), the outcome in the strain AT4@M9 (harboring AT4 both in the native position and also in M9), showed a multitude of structural analogues; various combinations of methyl, ethyl and allyl side chains at positions C21 and C11. This includes several novel structural analogues of FK506/FK520 (Fig. S11). Despite the fact that the use of multiple promiscuous AT domains in a single PKS results in significantly more challenging analytics and interpretation of data in general, the approach, combined with the use of super-producer chassis, allows detection of several new analogues in one go. Some of them would otherwise likely remain obscure.

## Methods

### Software, sequence analysis and bioinformatics methods

Genomic DNA from *S. ascomycinicus* H076 was extracted from early stationary phase cultures using the Nikodinovic method [47]. Genome sequencing was performed on the PacBio Sequel platform with sequencing library preparation using the SMRTbell template prep kit and SequelTM Binding Kit 2.0. Sequence reads were de novo assembled using HGAP2 software (Pacific Biosciences, Menlo Park, CA USA). Gene calling was performed using Prodigal software [48] and natural products pathways were annotated using antiSMASH 3.0 [49]. AntiSMASH was also used for the analysis of catalytic domain structure in the FK520 PKS. Multiple alignments of the PKS modules were performed using MUSCLE [50]. The newly sequenced FK520 BGC was compared to the deposited *S. ascomycinicus* ATCC 14,891 BGC sequence (GeneBank accession AF235504). 99.97% nucleotide identity was found across the BGC and 99.99% in the PKS genes (data not shown). Genetic design of constructs was performed using Geneious software (Biomatters LTD., Auckland, NZ).

### Molecular methods

AT domain exchange was achieved with double cross-over recombination, guided by an editing template. A classic positive/negative selection approach (*aac(3)IV* and *codA*) with a conjugative suicide vector was used [32]. The editing template was delivered on the same plasmid and included roughly 1000 bp-long homology flanks to support homologous recombination. The details on homology flanks for the constructs are given as positions relative to the sequence of *S. ascomycinicus* ATCC14891 BGC (GeneBank accession AF235504, Table S1). The genome editing was performed on an industrial producer *S. ascomycinicus* H076, derived from ATCC14891, however due to the high identity across the FK520 BGC, editing templates match both sequences. The flanks were interspaced with the replacement AT4 domain derived from the same sequence (GeneBank accession AF235504, Table S1). The editing template and the replacement AT domain were obtained synthetically from Genewiz, USA.

The completed vectors were introduced into *S. ascomycinicus* by conjugal transfer from *E. coli* ET12567 pUZ8002 using well-established methods [51]. Exoconjugants were selected for resistance to 60 mg L^− 1^ apramycin and subjected to selection for loss of the plasmid backbone (*codA* gene) in the presence of 5-fluorocytosine (50 mg L^− 1^) [32]. After the loss of all markers, colonies were screened by PCR to distinguish between WT revertants and AT domain replacement mutants. Details on PCR confirmation of genotypes are shown in Fig. S7 and Table S2.

Allylmalonyl-CoA supply genes *allA*, *allK*, *allR* and *allD* were obtained by DNA synthesis (Genewiz, USA), based on 6263 bp genomic region between positions 606,645 and 612,908 in reference to the *S. tsukubensis* NRRL 18488 genome (NCBI Bioproject: PRJNA382016). The synthetic fragment was then assembled with the integrative pSET152 plasmid [52] using the introduced *NdeI* (5’, in-frame) and *XbaI* (3’) restriction sites. The whole *allAKRD* operon is therefore under control of the strong ermE* promoter (permE*).

### Bacterial cultures

*Streptomyces ascomycinicus* (formerly *S. hygroscopicus var. ascomyceticus*) DSM 40,822 (ATCC 14891) and its derivative, a proprietary FK520 high-producer *S. hygroscopicus* H076, were used in this study. All manipulation on solid media was done with the TAA4 agar plates [21] (Table S3), incubated at 28 °C for 9 days. Where appropriate, apramycin (60 mg L^− 1^) and fluorocytosine (FC; 50 mg L^− 1^) were supplemented to the medium.

Liquid cultures were grown as described by Mo and Yang [53] with some modifications: Seed cultures were inoculated from a patch of confluent sporulating culture on TAA4 plates (2 × 2 cm) into 250 mL Erlenmeyer flasks with 50 mL of seed medium (Table S4). Cultures were then incubated on a rotary shaker at 28 °C and 180 rpm for 41 h. One mL of seed culture was transferred to 100 mL Erlenmeyer flask containing 15 mL of main culture media (Table S5). Where indicated, L-Isoleucine, L-Leucine or L-Valine was added to the medium before sterilization. Cultures were incubated on a rotary shaker at 26 °C, 260 rpm (2.5 cm throw) for 5 days and sampled periodically. For purposes of compound isolation, cultivation was linearly scaled up to 1.6 L culture volume. 2 L bench scale fermentors (Sartorius) with double Rushton impeller configuration were used at atmospheric pressure. No pH control or nutrient feeding was used, while the stirring and airflow were kept constant (1200 rpm and 0.55 VVM, respectively). dO_2_ levels reached between 40 and 50% at maximum oxygen demand. Harvests were performed after 160–210 h of cultivation.

### HPLC-MS analytics of cultures

The polyketide products were measured with the following method: 5 g of culture broth was diluted with 10 mL of MeCN/H_2_O = 9:1 and pH adjusted to 5. The mixture was stirred for 20 min at 720 rpm and then centrifuged (20 min, 15000 × g, 4 °C). 5 µL of supernatant was injected onto a Phenomenex Kinetex C18 column (150 × 2.1 mm, 1.7 μm) with a flow rate of 0.25 mL min^− 1^ and a column temperature of 60 °C. Mobile phase A (MPA; H_2_O/MeCN/MTBE/HCOOH = 600:330:70:1) and mobile phase B (MPB; H_2_O/MeCN/MTBE/HCOOH = 100:830:70:1) were used. MPA was supplemented with 0.4 mM NH_4_HCO_2_. The method started with an isocratic hold at 100% MPA for 30 min, followed by a 10 min linear gradient to 90% MPB and a 10 min re-equilibration to starting conditions. An LCQ ion trap mass spectrometer (ThermoFisher, USA) equipped with a HESI source was used for MS detection. Positive ionization (source voltage 4 kV, capillary temperature 275 °C, capillary voltage 42.5 V, sheath gas 10 AU, auxiliary gas 5 AU, sweep gas 0 AU) and full-scan monitoring in the *m/z* range of 300–1000 enabled the detection of the compounds. UV diode array multi-wavelength detection was also performed, primarily to calculate concentrations of compounds **4**, **5**, **6**, and **7** at 210 nm.

Intracellular acyl-CoA metabolites were measured with a modified method of Gläser et al. [54]. The modification included the use of Syncronis aQ (100 mm × 2.1 mm, 1.7 μm, ThermoFisher) column, as well as a slight modification of the mobile phase gradient where the method started with a 12 min linear gradient from 0 to 15% MPB, continued with a steep 2 min gradient to 100% MPB, an isocratic hold at 100% MPB for 2 min and a 2 min gradient back to starting conditions of 100% MPA. The column then re-equilibrated for 5 min before the next injection. A TSQ Quantum Access MAX mass spectrometer (ThermoFisher, USA) equipped with a HESI source was used for the detection, using single reaction monitoring (SRM). The following SRM transitions were monitored: propionyl-CoA (m/z [M + H]^+^ = 824.6 → 317.1); butytyl/isobutyryl-CoA (m/z [M + H]^+^ = 838.1 → 331.1); ethylmalonyl-CoA (emal; m/z [M + H]^+^ = 882.1 → 375.1) and methylmalonyl-CoA (mmal; m/z [M + H]^+^ = 868.0 → 361.1). Mass spectrometer settings were: spray voltage 4 kV, vaporizer temperature 50 °C, sheath gas pressure 53 AU, ion sweep gas pressure 0 AU, auxiliary gas pressure 4 AU, capillary temperature 275 °C, collision energy 30 V.

### Purification and structure confirmation of compounds

The fermentation broth was harvested after 160–210 h of cultivation. pH was adjusted to 7 with NaOH and extracted with 1 vol. of toluene. The extraction was repeated two more times with 0.5 vol. toluene. Collected organic phases (~ 2 L) were polished twice with 100 mL of water. The solvent in toluene phases was exchanged to MeOH by evaporation to yield ~ 100 g L^− 1^ solutions of **4**, **5**, **6**, and **7**. For defatting, 1 vol. of petrol ether was added to the solutions and phases separated by drop-vise addition of water up to 0.5 vol. of initial solutions. This was repeated twice with recovery of the MeOH/water phase. To increase the recovery yield, the collected petrol ether fractions were re-extracted with 0.3 vol. of MeOH/water (2:1). The MeOH/water fractions were collected and evaporated to dryness. Samples were dissolved in MeOH to achieve concentrations of 50–200 mg mL^− 1^. 4 mL of the sample was injected onto a Thermo Syncronis C18 column (250 × 21.2 mm, 5 μm) with a column temperature of 60 °C.

Method 1: For purification of **5**, **6**, and **7**, mobile phase A (MPA; H_2_O/MeCN = 600:400) and mobile phase B (MPB; H_2_O/MeCN = 200:800), both supplemented with 0.1% formic acid were used with a flow of 27 mL min^− 1^. The method started with a 60 min linear gradient from 44% MPB to 48% MPB followed by a 20 min gradient ending in 100% MPB. Conditions were then held at 100% MPB for 20 min, followed by a final 10 min re-equilibration step to starting conditions.

Method 2: For the purification of **4**, mobile phase A (MPA; H_2_O/MeCN/MTBE = 630:300:70) and mobile phase B (MPB; H_2_O/MeCN/MTBE = 230:700:70), both supplemented with 0.1% formic acid were used with a flow rate of 22 mL min^− 1^ at 0% MPB for the first 50 min. This was followed by a fast 5 min linear gradient to 100% MPB, a 15 min holding step and concluded with a final 15 min re-equilibration step.

In all cases, 45 mL fractions were collected and analyzed. Fractions containing the desired products underwent solid-phase extraction using Amberlite XAD 1600 N resin. MTBE and MeOH were used sequentially to elute the products from the resin.

After evaporation of solvents, the material (white crystals) was dissolved in 0.7 mL of CDCI; (D, 99.95%, Merck) and characterized by ^1^H and ^13^C NMR on a Bruker Avance III HD spectrometer operating at 600 and 150 MHz for ^1^H and ^13^C NMR, respectively. The spectrometer was equipped with a 5 mm BBO, Z-gradient probe. Spectra were acquired using Bruker TopSpin software (ver. 3.6) and processed using MesReNova software (ver. 15.1). The compounds are present in two isomers, chemical shifts are listed for the major and minor isomer, respectively. Chemical shifts (δ) are expressed in ppm with reference to residual solvent signal (7.26 ppm and 77.0 ppm for ^1^H and ^13^C NMR, respectively). 2D correlations were performed with COSY, HSQC, HMBC and NOESY spectra.

### Chlorination and final purification

350 mg of either **4**, **5**, **6**, or **7** was transferred to a round-bottom flask. Toluene (100 mL) was added and distilled off twice to remove the residual water. The remaining solid was dissolved in anhydrous THF, which was then again distilled off and replaced with fresh anhydrous THF (2.75 mL). A fresh stock of chlorination reagent was prepared as follows (quantities are given per reaction): In an Erlenmeyer flask, triphenylphosphine (two-fold molar excess) was dissolved in anhydrous THF (2.75 mL). N-chlorosuccinimide (NCS) was added to this solution in portions, to yield a 2.33-fold molar excess. This reaction mixture was stirred at room temperature, followed by addition of S-collidine (4-fold molar excess, 0.255 mL). The THF solutions of compounds **4**, **5**, **6** and **7** were individually placed into 15 mL reaction tubes, each combined with the chlorination reagent (3 mL), and sealed. The reaction tubes were placed in an orbital incubator, and stirred at 220 rpm and 42 °C. After 20 h, the reactions were quenched. A 1.5 mL mixture of water, MeOH and citric acid, in a ratio of 8:6:1 was added to the reaction mixture and the products were extracted twice with cyclohexane (2 × 30 mL). Organic phase was collected and evaporated to dryness. The resulting compound 8 was subjected to preparative HPLC using the Method 1 conditions described above. For purification of **9**, **10** and **11**, Method 1 was modified so that it started with a 21.5 min linear gradient from 56% to 57% MPB, followed by a 118 min isocratic step. This was followed by a 10 min linear gradient to 100% MPB, and holding at 100% MPB for 10 min. The run was finished with a 10 min re-equilibration step to initial conditions.

LC-MS-UV analysis was done by injecting 5 µL of samples (prepared in either 67% acetonitrile or 100% methanol, preparative fractions were injected directly) onto Phenomenex Kinetex C18 column (100 × 2.1 mm, 1.7 μm) with a flow rate of 0.9 mL min^− 1^ and a column temperature of 65 °C. Mobile phase A (MPA; H_2_O/MeCN/MTBE/HCOOH = 630:300:70:1) and mobile phase B (MPB; H_2_O/MeCN/MTBE/HCOOH = 230:700:70:1) were used for the gradient program, starting with an isocratic hold at 20% MPB for 10.5 min. Next, a 1 min gradient to 50% MPB with a 0.5 min hold was followed by another 1 min gradient to 100% MPB and then a steep return to starting conditions at 20% MPB with a 2 min hold. UV diode array multi-wavelength detection was performed to calculate concentrations and purities of compounds at 210 nm. The mobile phase flow was then split with a 1:5 splitter and the low flow was introduced into an LCQ ion trap mass spectrometer (ThermoFisher, USA) equipped with a HESI source for MS detection. Positive ionization (source voltage 4 kV, capillary temperature 275 °C, capillary voltage 42.5 V, sheath gas 10 AU, auxiliary gas 6 AU, sweep gas 0 AU) and full-scan monitoring in the selected *m/z* range enabled the detection of the compounds.

## Supplementary Information

Supplementary Material 1.

Supplementary Material 2.

## Data Availability

Data generated or analyzed during this study are included in this published article and its supplementary information files. Plasmids used in this study are available upon request. The strain **S. ascomycinicus** H076 and its mutants can only be shared under MTA upon consideration of specific cases, however the work can be reproduced with publicly available **S. ascomycinicus** DSM 40822 (ATCC 14891), as indicated in the article. Sequence data for the FK520 biosynthetic cluster are not shown, however 99.97% nucleotide identity was found across the BGC and 99.99% in the PKS genes compared to the GeneBank accession AF235504.
