# Supplementary material for: Engineering of FK520 polyketide synthase for rapid access to quality control reference standards
Source: Microb Cell Fact. 2025 Nov 28;24:243. doi: 10.1186/s12934-025-02861-3 (PMC12664185; doi:10.1186/s12934-025-02861-3)
Supplement: Supplementary file 1 — Supplementary Material 1. [file 12934_2025_2861_MOESM1_ESM.docx]

Supplementary Information 1 for:

**Engineering of FK520 Polyketide Synthase for Rapid Access to Quality Control Reference Standards**

Nina Žigart, Petra Pivk Lukančič, Tjaša Drčar, Jan Peterka, Maja Harej Perko and Peter Mrak*

*peter.mrak@sandoz.com

**This PDF file includes:**

- Figs. S1 to S12
- Tables S1 to S6
- Information S1


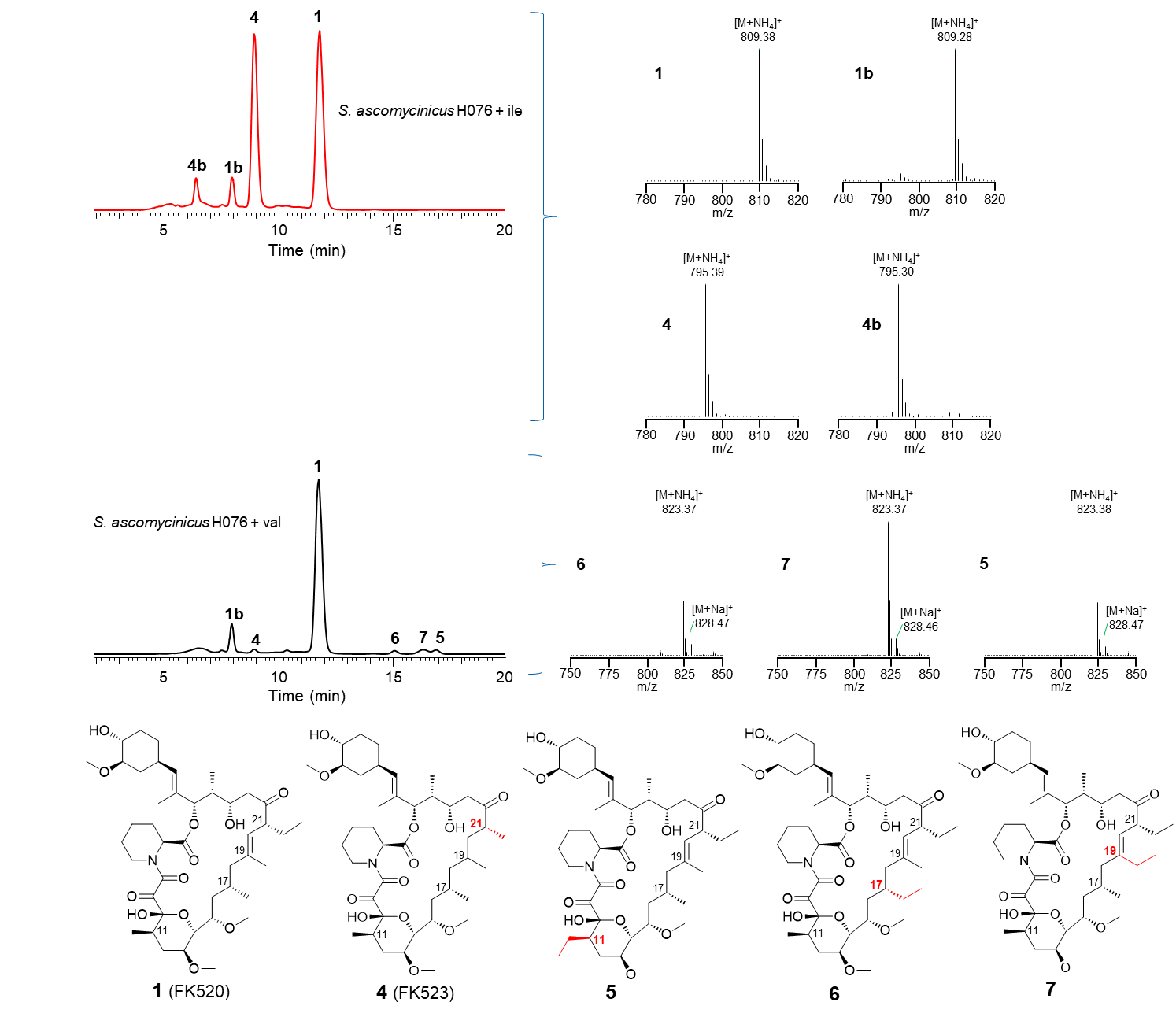


Fig. S1 MS (ESI+) spectra of structural analogues found in cultures of *S. ascomycinicus*. Chromatograms from Fig. 2 are the source of the data.


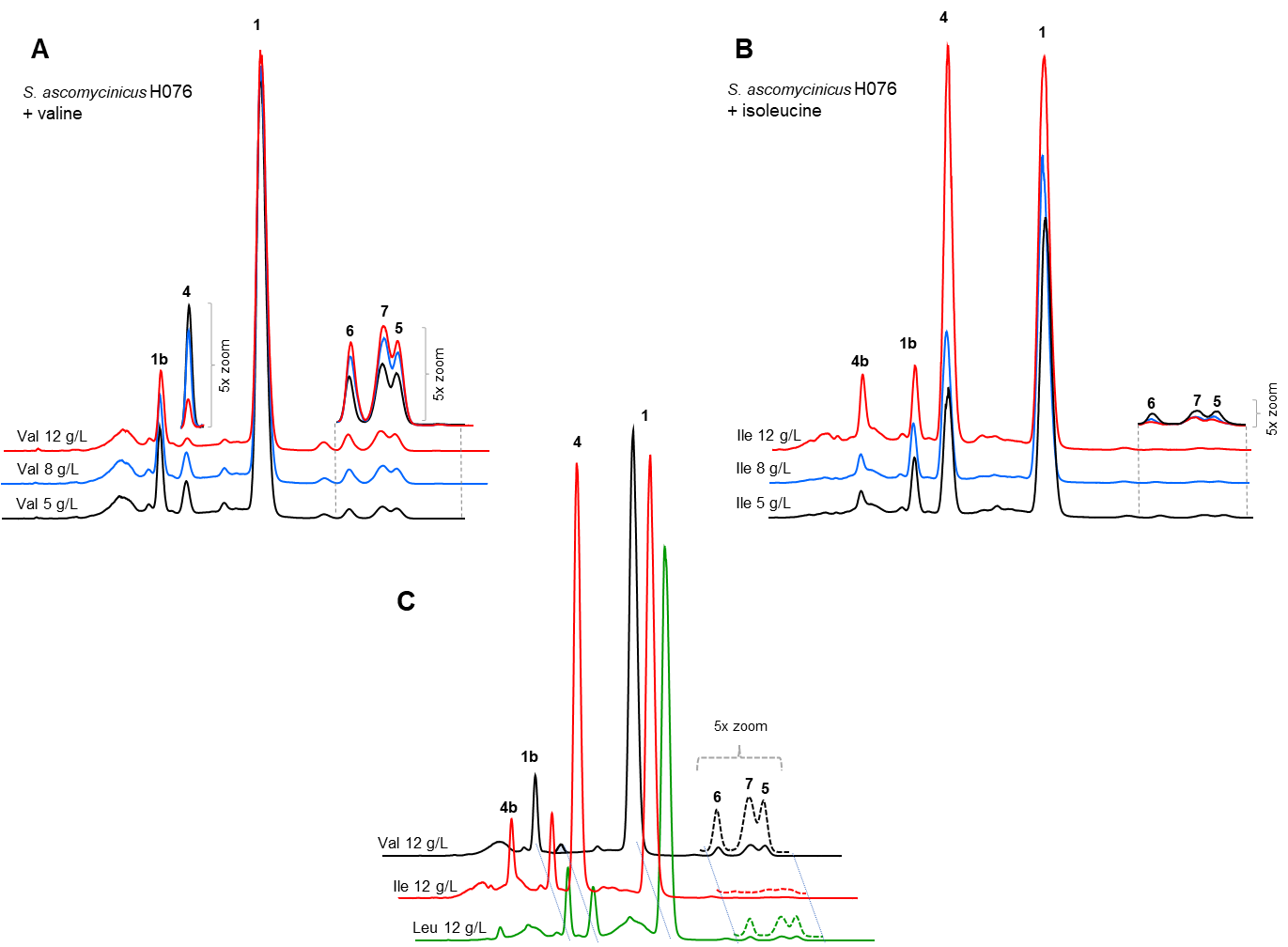


Fig. S2 Effects of an addition of valine, isoleucine and leucine to cultures of *S. ascomycinicus* H076. (A) Dose dependent response of *S. ascomycinicus* cultures to the addition of valine. Extracted LC-MS chromatograms (ESI+, *m/z* = 795, 809, 823) in the time frame of 2 to 20 min are shown. For clarity, sections of chromatograms with 4, 5, 6 and 7 are zoomed in. (B) A dose dependent response of *S. ascomycinicus* cultures to the addition of isoleucine. Extracted LC-MS chromatograms (ESI+, *m/z* = 795, 809, 823) in the time frame of 2 to 20 min are shown. For clarity, sections of chromatograms with 5, 6 and 7 are zoomed in. (C) Comparison of the effects of the addition of valine, isoleucine and leucine (12 g L^-1^) to cultures of *S. ascomycinicus* H076.

NH

2

O

OH

**L-valine**

NH

2

O

OH

**L-isoleucine**

S-CoA

O

isobutyryl-CoA

S-CoA

O

**butyryl-CoA**

**(2S)-methylmalonyl-CoA**

**(mmal)**

HO

S-CoA

O

O

S-CoA

O

**crotonyl-CoA**

S-CoA

O

hydroxybutyryl-CoA

OH

**(2S)-ethylmalonyl-CoA**

**(emal)**

HO

S-CoA

O

O

S-CoA

O

acetoacetyl-CoA

O

O

O

n

polyhydroxybutyrate

S-CoA

O

2-methylbutyryl-CoA

S-CoA

O

2-methylacetoacetyl-CoA

O

S-CoA

O

tiglyl-CoA

S-CoA

O

**propionyl-CoA**

S-CoA

O

succinyl-CoA

HO

O

(2R)-methylmalonyl-CoA

HO

S-CoA

O

O

S-CoA

O

methylacrylyl-CoA

S-CoA

O

β

-hydroxyisobutyryl-CoA

HO

S-CoA

O

(2S)-methylmalonyl-CoA

semialdehyde

H

O

OH

O

(2S)

β

-hydroxyisobutyrate

HO

OH

O

(2S)-methylmalonate

semialdehyde

H

O

ICM

PCC?

CCR

(FkbS)

FkbE

MCR

MCM

S-CoA

O

methylsuccinyl-CoA

HO

O

ECM

FkbU

isoleucine catabolism

butyryl-CoA pathway

isoleucine catabolism

BGC-encoded crotonyl-CoA pathway

ECM pathway

MCM pathway

Fig. S3 The metabolic pathways contributing to methylmalonyl-CoA (mmal) and ethylmalonyl -CoA (mmal) pools. The Val to emal pathway (butyryl-CoA pathway) and Ile to mmal pathway (isoleucine catabolic pathway to propionyl-CoA) are most likely explanation for the behavior observed with cultures of *S. ascomycinicus*. This scheme was derived from multiple sources. [26, 27, 28, 29, 30]


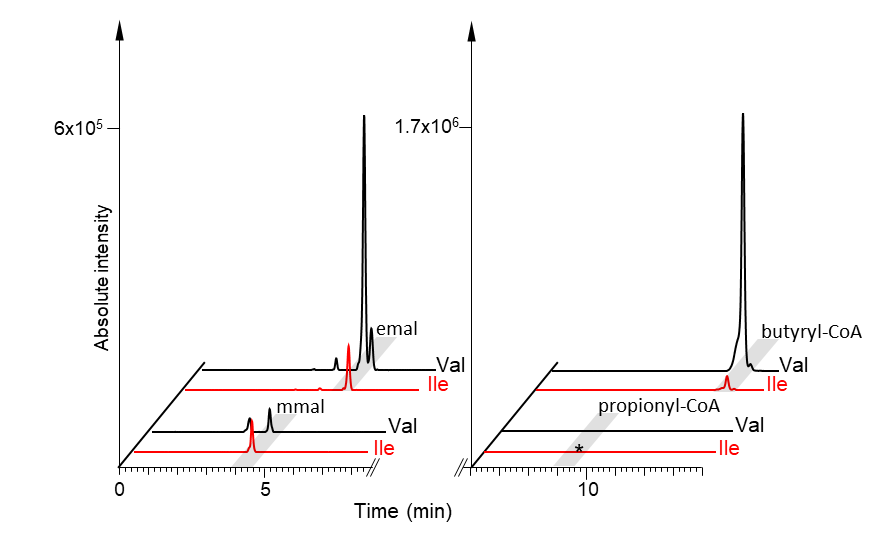


Fig. S4 LC-MS chromatograms showing SRM transitions for propionyl-CoA (m/z M+H^+^= 824.6 → 317.1), butytyl/isobutyryl-CoA (m/z M+H^+^= 838.1 → 331.1), ethylmalonyl-CoA (emal; m/z M+H^+^= 882.1 → 375.1) and methylmalonyl-CoA (mmal; m/z M+H^+^= 868.0 → 361.1). Representative chromatograms for 3-day-old *S. ascomycinicus* H076 cultures grown with Isoleucine or Valine are shown (3 independent samples were analyzed for each on various days). *Propionyl-CoA could not be reliably detected, however sporadic samples from cultures with Ile did show propionyl-CoA peak on the limit of detection.


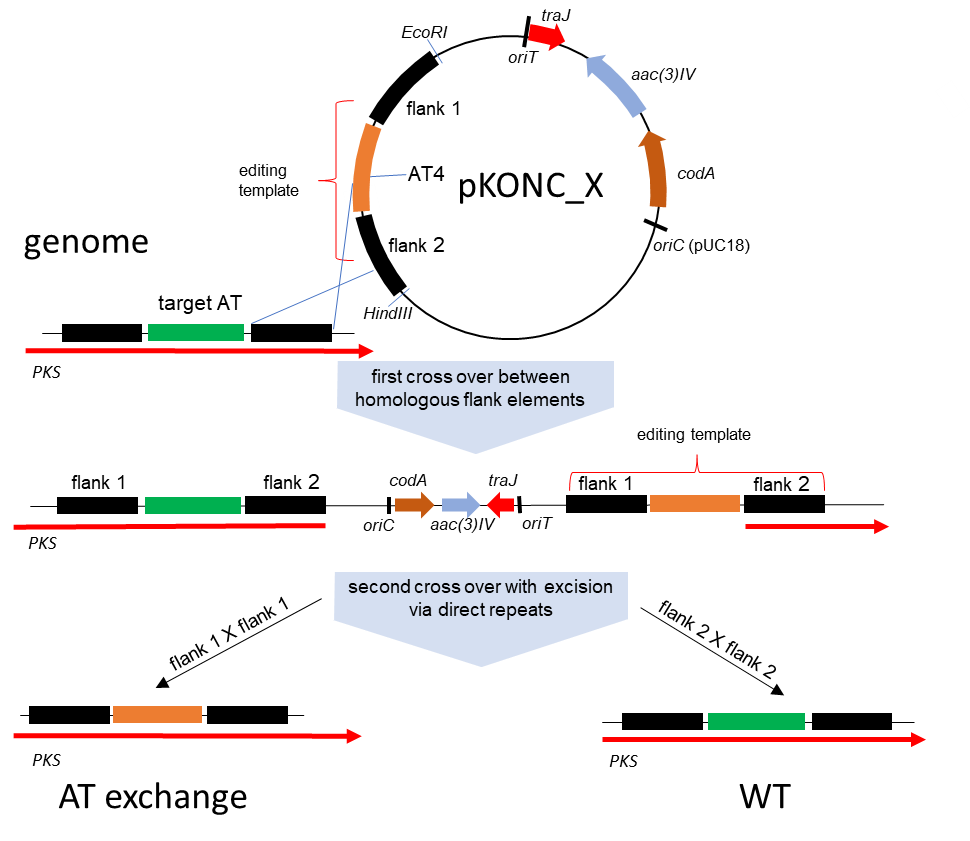


Fig. S5 The genome editing system used in this study. Map of the editing vector pKONC containing positive / negative selection markers and an editing template. The recombination outcomes after first and second cross-over are shown. The resulting marker-less colonies have either the desired AT exchange or revert back to WT. Screening with colony PCR allows identification of the latter.

**Table S1 Details on the editing templates used in this study.** Editing template was composed of flank 1 and flank 2, interspaced with the replacement AT domain. The length and position of each segment according to the deposited *S. ascomycinicus ATCC14891 FK520 BGC (GeneBank accession AF235504)* is shown. The individual segments were obtained by DNA synthesis (Genewiz, USA), assembled in the following order: EcoRI-flank1-AT4-flank2-*HindIII* and cloned into *EcoRI*, *HindIII* restriction sites of the plasmid backbone.

| **Construct** | **flank 1**  **(position)** | **AT domain replacement**  **(position)** | **flank 2**  **(position)** |
| --- | --- | --- | --- |
| pKONC_AT4@M5 | 1000 bp  (23390 - 22391) | 843 bp  (22390 - 21548) | 1000 bp  (21547 - 20548) |
| pKONC_AT4@M6 | 1000 bp  (18557 - 17558) | 843 bp  (17557- 16715) | 1000 bp  (16714 - 15715) |
| pKONC_AT4@M9 | 1000 bp  (63114 - 64113) | 843 bp  (64114 - 64956) | 1000 bp  (64957 - 65956) |


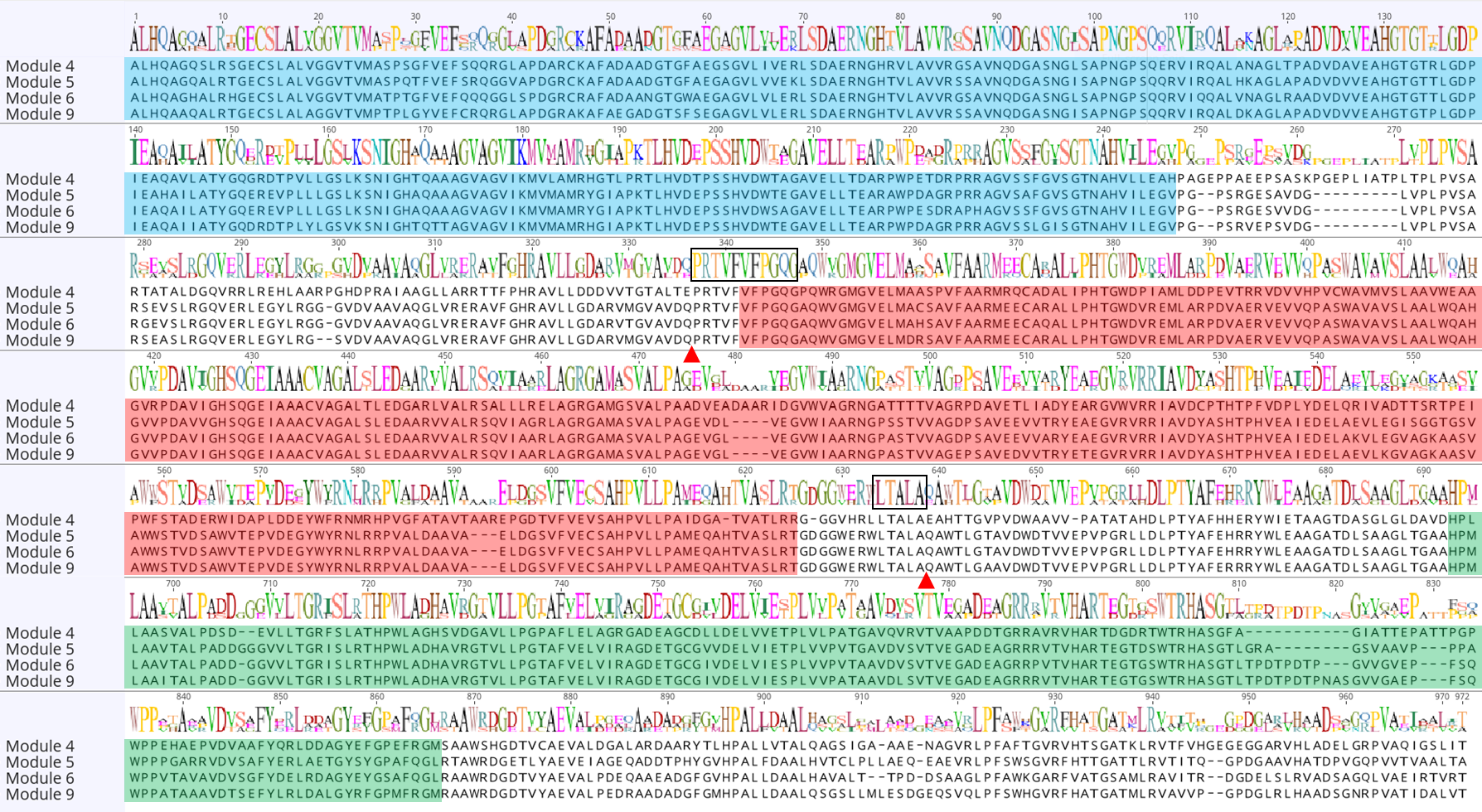


**Fig. S6** Multiple alignment of AT domain-containing section of modules 4, 5, 6 and 9 of the FK520 PKS with highlighted domains (KS domain – blue, AT domain – red, DH domain – green). The conserved amino acid motifs on the AT domain borders are boxed. Exact points of the AT domain exchanges are marked with the red arrow.

**Table S2 List of confirmation PCR primers and expected amplicons for WT and the mutant genotypes**. The PCR reaction was carried out with Q5 High-Fidelity DNA Polymerase (New England Biolabs). 25 μL of the reaction mixture was composed of: Q5 Reaction Buffer (1×), 200 μM dNTP, 0.5 μM of each primer, Q5 High GC Enhancer (1×), approximately 50 ng of template DNA and 0.5 unit of enzyme. The thermal profile started with a denaturation step at 94 °C for 5 min. The thermal cycle for the following 30 cycles was 94 °C for 20 s (denaturation step), 62 °C for 20 s (annealing step), and 72 °C 45 s kb**^-1^** (extension step). The final elongation step was carried out at 72 °C for 7 min.

| **Mutant** | **Primer pair** | **Expected amplicon** | |
| --- | --- | --- | --- |
|  |  | WT | mutant |
| **AT4@AT5** | 1. CGTCCTCGTCGTCGAAAAGCTCTC ; GTACCGCGTCACCACTTCTTCCAC  2. CGTCCTCGTCGTCGAAAAGCTCTC ; GACGATCCGCTGGAGTTCGTCG | 1306 bp  none | none  1416 bp |
| **AT4@AT6** | 3. GTTGGTGAACGCCGGACTGC ; GCACGCCTTCGGCCTCATAC  4. GTTGGTGAACGCCGGACTGC ; GACGATCCGCTGGAGTTCGTCG | 1172 bp  none | none  1266 bp |
| **AT4@AT9** | 5. CACACCGCTCTACCTCGGTTCG ; CACGCCTTCGGTCTCATACCG  6. CACACCGCTCTACCTCGGTTCG ; GACGATCCGCTGGAGTTCGTCG | 1045 bp  none | none  1138 bp |


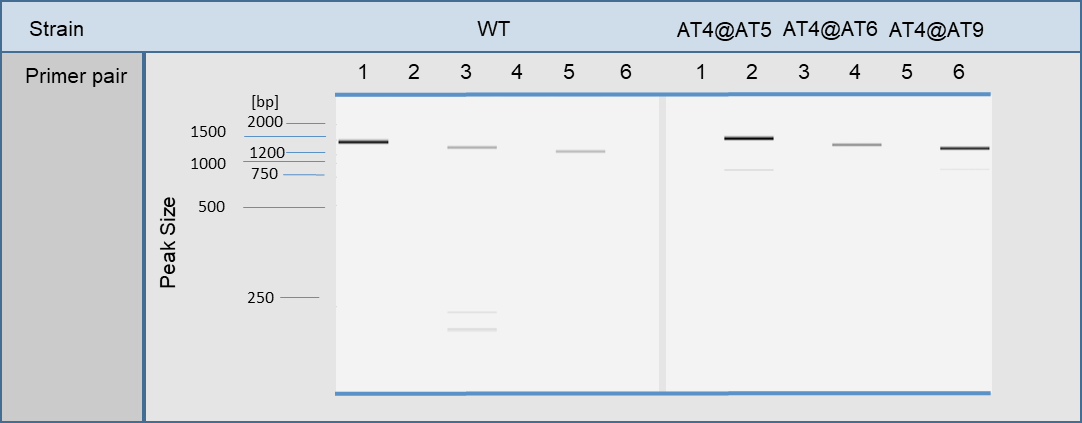


**Fig. S7 Electropherograms of genotype confirmation PCRs.** Capillary gel electrophoresis was used to analyze the PCR reactions. List of primers, expected amplicon length, and PCR conditions is given in Table S2.


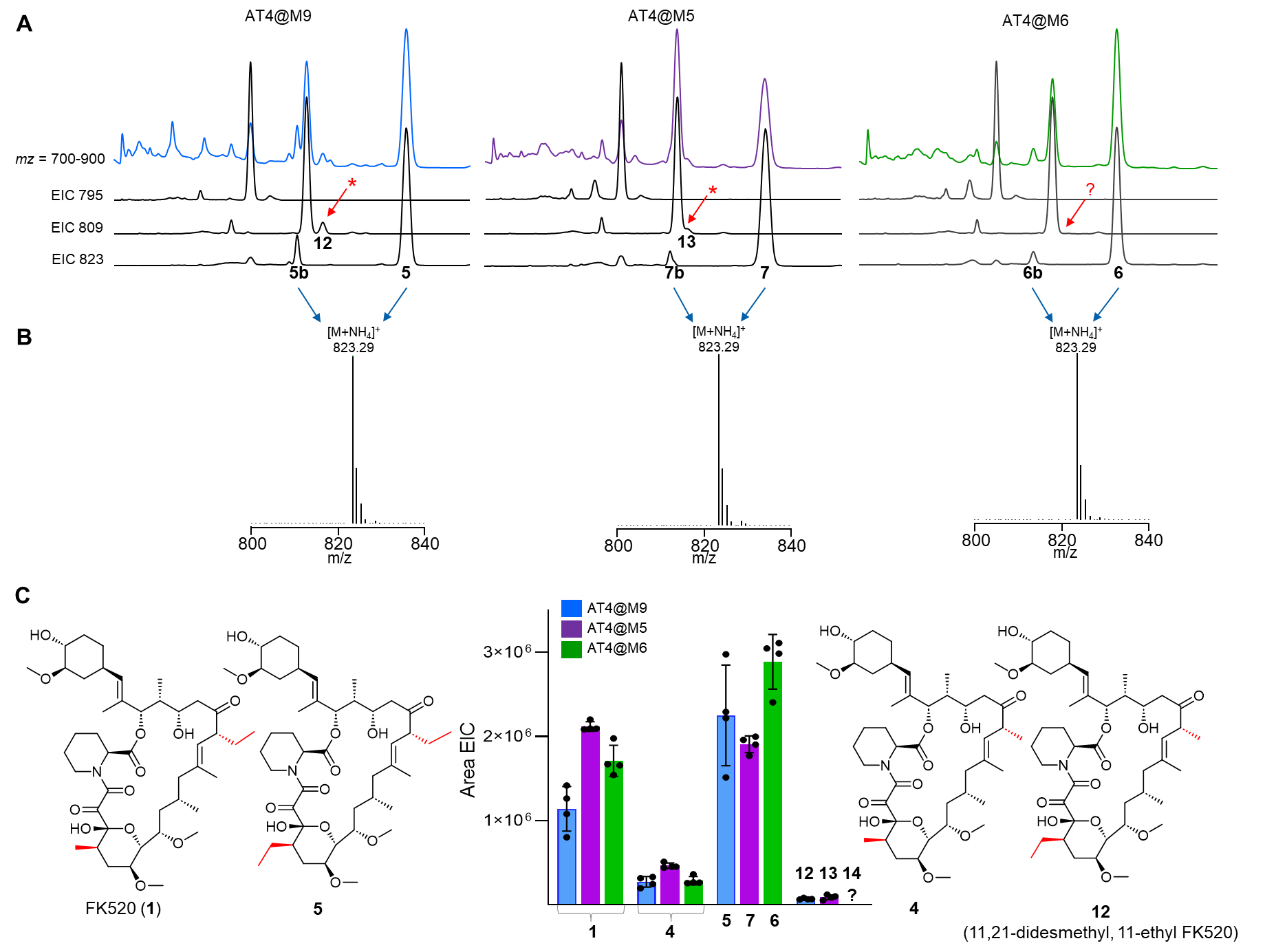


Fig. S8 (A) Extracted chromatograms (ESI+, *m/z* = 795, 809 and 823) of AT4@M9, AT4@M5 and AT4@M6 mutants supplemented with isoleucine. The time frame of 2 to 20 min is shown. The minor analogues 12 and 13 are marked with a red arrow. (B) MS spectra of structural analogues 5, 6 and 7 and their equilibrium isomers in the AT replacement mutants. In all cases the spectra agree with the presence of an additional CH_2_ moiety (*m/z* +14; -CH_3_ to -CH_2_-CH_3_). (C) Structures of the four distinct structural analogues produced by AT4@M9 under conditions of increased mmal pool (cultures supplemented with Ile). The product distribution in 4 independent experiments is shown in the bar chart for AT4@M9, AT4@M5 and AT4@M6. Data are specific EIC (± 0.5 Da) peak areas for each compound (n = 4). We predict that a compound analogous with 12 and 13, is accumulating also in AT4@M6 but is chromatographically inseparable from 1. Therefore, full distribution data for this strain could not be obtained.

Info S1 Statistical analysis of the PKS product distribution for the engineered PKS strains cultivated in the presence of isoleucine.

1. AT4@M9 engineered PKS strain contains AT4 in modules M4 and M9.

The product distribution data shown in Fig. S6C was used to prepare a contingency table for AT4@M9. The table data are specific EIC peak areas for each compound from the 4 experiments for each strain. GraphPad Prism 10.3.0 was used to perform statistical analysis.

|  | side chain | | area EIC | | | | | | distribution | |
| --- | --- | --- | --- | --- | --- | --- | --- | --- | --- | --- |
| compound | C21 | C11 | exp. 1 | | exp. 2 | exp. 3 | exp. 4 | sum | found | expected |
| 1 | ethyl | methyl | 801843 | 1262443 | | 1416775 | 1076476 | 4557537 | 31.17% | 24% |
| 4 | methyl | methyl | 193479 | 314902 | | 329470 | 244667 | 1082518 | 7.52% | 16% |
| 5 | ethyl | ethyl | 1512443 | 2292082 | | 2976847 | 2217865 | 8999237 | 58.79% | 36% |
| 12 | methyl | ethyl | 64951 | 60132 | | 63756 | 79475 | 268314 | 2.53% | 24% |

Chi-square goodness of fit was used to test the null hypothesis that distribution of incorporated extenders with the AT4 domain in the system is 0.6 for emal and 0.4 for mmal for both positions. This hypothesis is based on data with WT strain grown in the presence of isoleucine (Fig. 2, Fig. 3), and gives the following expected distribution: 1: 24%, 4: 16%, 5: 36% and 12: 24%. The chi-square analysis at 3 DF, shows P < 0.0001 for each of the experiments as well as for sum of counts across the experiments. While the observed distribution could be attributed to general bias toward emal, this is disturbed by low counts on 12, suggesting interdependence in operation of the two AT4s.

The interdependence between AT4@M4 and AT4@M9 was tested by forming a 2×2 contingency table for the sum of counts across the experiments. Chi-square with Yates’ correction was used to calculate the odds ratio. The results show that the AT4@M9 is 6.343 times more likely to integrate emal (rather than mmal) if emal was already incorporated by AT4@M4 with P < 0.0001. To confirm that the variance in distribution between the 4 independent experiments does not impact the overall conclusion on the biases, the procedure was repeated on separate contingency tables for each individual experiment. In all cases the chi-square tests confirm the conclusions with P < 0.0001.

The observed extender proportions (^a^ percent emal integration by the module) and odds ratios (^b^ relative likelihood of emal integration at C11 when ethyl was incorporated at C21) for each individual experiment are listed in the table below:

|  | exp. 1 | | exp. 2 | | exp. 3 | | | exp. 4 | |
| --- | --- | --- | --- | --- | --- | --- | --- | --- | --- |
|  | ^a^ emal % | ^b^ emal odds | ^a^ emal % | ^b^ emal odds | ^a^ emal % | ^b^ emal odds | ^a^ emal % | | ^b^ emal odds |
| AT4@M4 | 89.96 | - | 90.46 | - | 91.79 | - | 91.04 | | - |
| AT4@M9 | 61.31 | 5.619 | 59.86 | 9.508 | 63.52 | 10.86 | 63.49 | | 6.343 |

1. AT4@M5 engineered PKS contains AT4 in modules M4 and M5.

The product distribution data shown in Fig. S6C was used to prepare a contingency table for AT4@M5. The table data are specific EIC peak area for each compound from the 4 experiments for each strain. GraphPad Prism 10.3.0 was used to perform statistical analysis.

|  | side chain | | area EIC | | | | | distribution | |
| --- | --- | --- | --- | --- | --- | --- | --- | --- | --- |
| compound | C21 | C19 | exp. 1 | exp. 2 | exp. 3 | exp. 4 | sum | found | expected |
| 1 | ethyl | methyl | 2093130 | 2091617 | 2082601 | 2201366 | 8468714 | 47.94% | 24% |
| 4 | methyl | methyl | 450778 | 449502 | 439133 | 509619 | 1849032 | 10.32% | 16% |
| 7 | ethyl | ethyl | 1770055 | 1902584 | 1995581 | 1958303 | 7626523 | 40.54% | 36% |
| 13 | methyl | ethyl | 52319 | 96512 | 119255 | 83969 | 352055 | 1.198% | 24% |

Statistical analysis was performed in the exact same manner as above, again supporting all conclusions both at the sum of counts as well as with individual experiments with P < 0.0001. Again, the expected distribution is disturbed by low counts on 13, indicating interdependence in the operation of the two AT4s.

Odds ratio (on the sum of counts) calculated through Chi-square with Yates’ correction shows that the AT4@M9 is 4.730 times more likely to integrate emal (rather than mmal) if emal was already incorporated by AT4@M4 with P < 0.0001.

The observed extender proportions (^a^ percent emal integration by the module) and odds ratios (^b^ relative likelihood of emal integration at C19 when ethyl was incorporated at C21) for each individual experiment are listed in the table below:

|  | exp. 1 | | exp. 2 | | exp. 3 | | | exp. 4 | |
| --- | --- | --- | --- | --- | --- | --- | --- | --- | --- |
|  | ^a^ emal % | ^b^ emal odds | ^a^ emal % | ^b^ emal odds | ^a^ emal % | ^b^ emal odds | ^a^ emal % | | ^b^ emal odds |
| AT4@M4 | 88.48 | - | 87.98 | - | 87.96 | - | 87.51 | | - |
| AT4@M5 | 41.47 | 7.286 | 44.04 | 4.237 | 45.61 | 3.528 | 42.97 | | 5.399 |


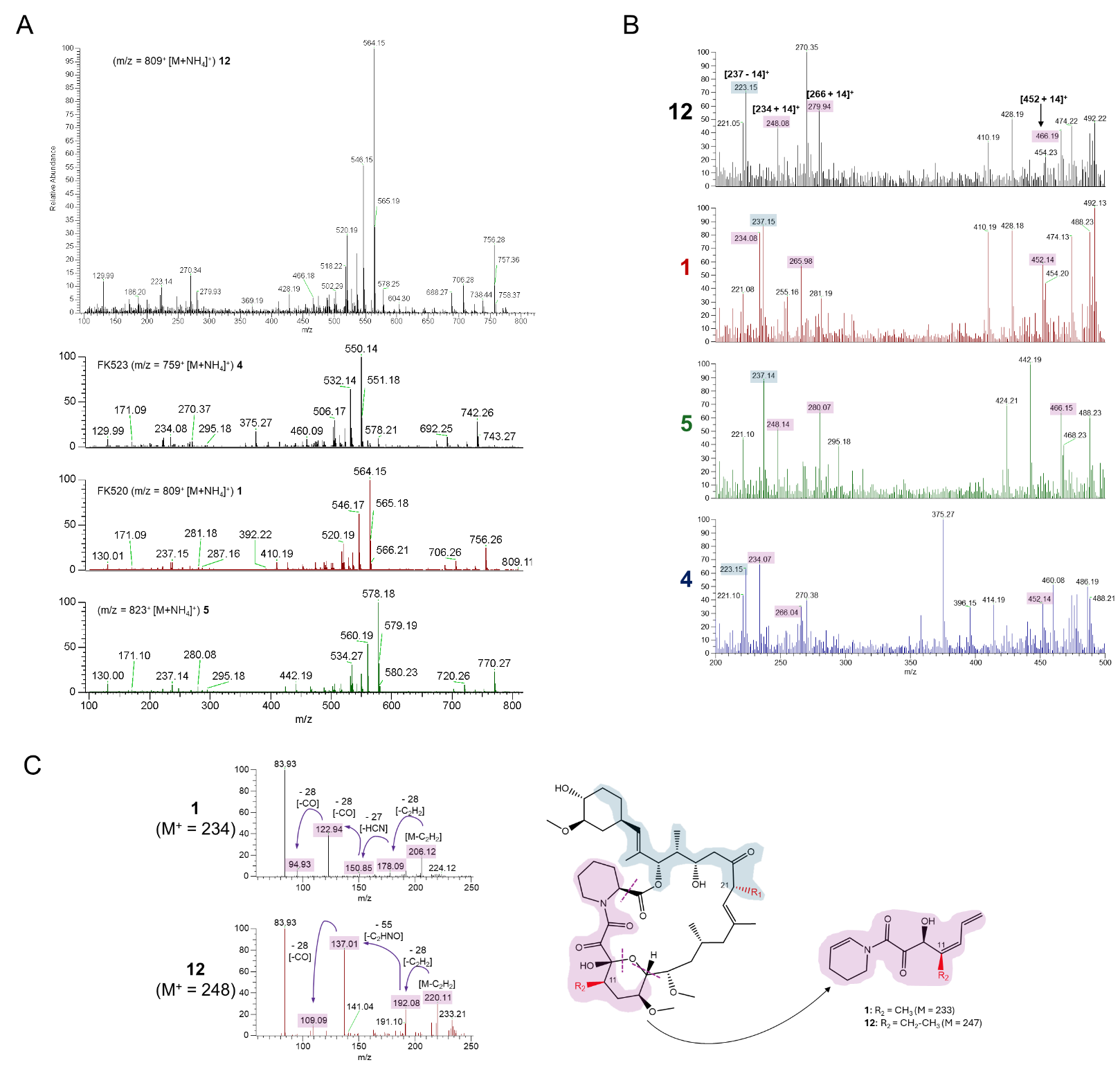


Fig. S9 Fragmentation spectra and patterns of 12 (*m/z* = 809^+^ [M+NH_4_]^+^) found in the AT4@M9 mutant supplemented with Ile and its comparison to 4, FK520 (1), and 5. (A) Fragmentation spectra of 12, 4, 1 and 5. (B) A part of the fragmentation spectra of 12 (black), FK520 (1, red), 5 (green), and 4 (blue), which was augmented to show the fragmentation specifics. Differences between 1 and 12, and corresponding Δ *m/z* are marked. Light purple markings indicate the presence of an ethyl side chain at C11 (found in 12 and 5) and methyl side chain at C11(found in 1 and 4). Light blue markings indicate the presence of the methyl side chain at C21 (found in 12 and 4) and the ethyl side chain at C21 (found in 1 and 5). (C) Fragmentation spectra of the fragment *m/z* = 234^+^ in FK520 (1) and *m/z* = 248^+^ in 12 with marked fragment losses. The FK506 core structure is shown, depicting the position of the two key fragments used for structural confirmation. The fragment containing position C11 (*m/z* = 234^+^ vs. 248^+^) is highlighted light purple, and the fragment containing position C21 (*m/z* = 223^+^ vs. 237^+^) is highlighted light blue.


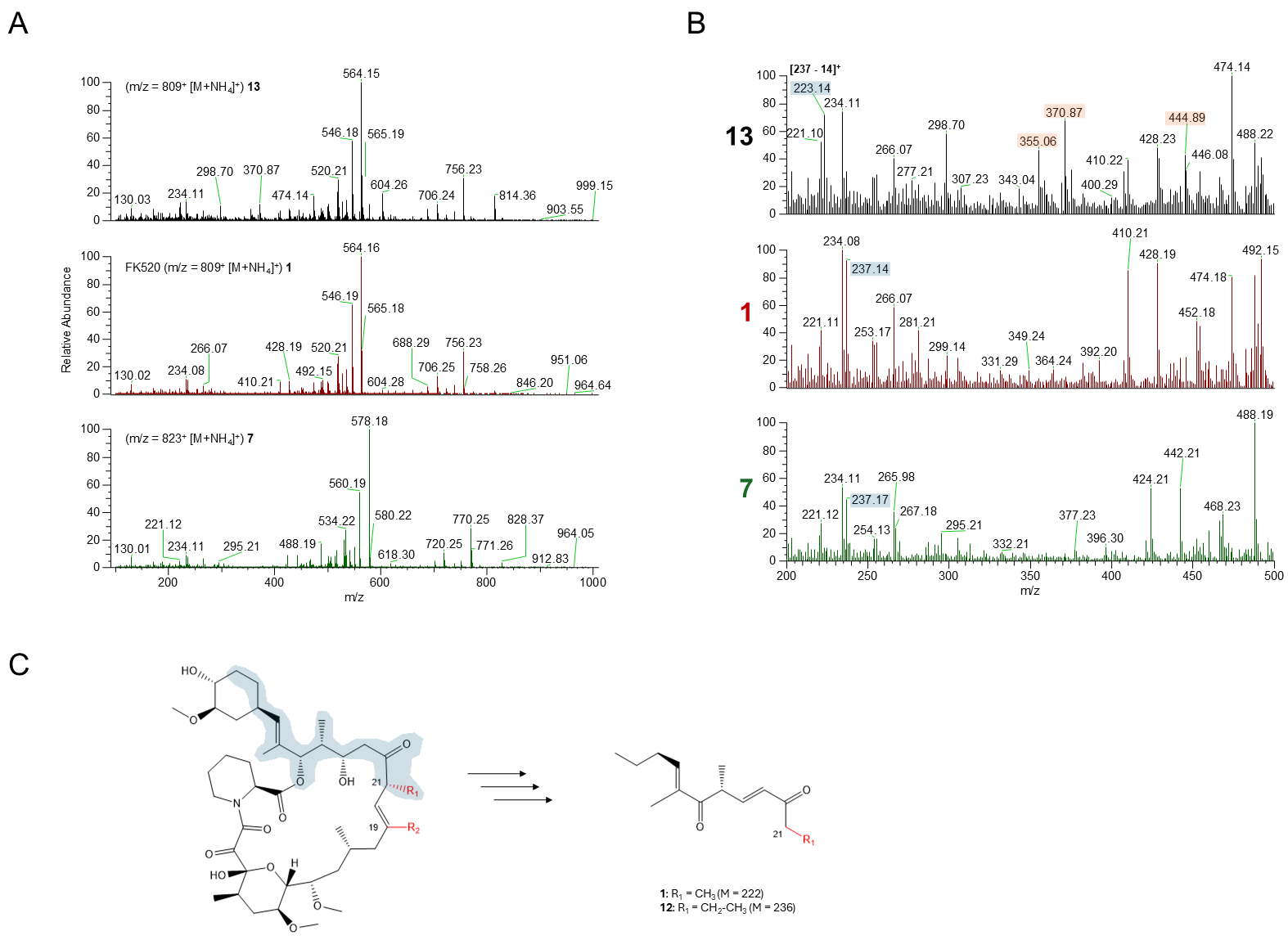


Fig. S10 Fragmentation spectra of 13 (*m/z* = 809^+^ [M+NH_4_]^+^) found in the AT4@M5 mutant supplemented with Ile and its comparison to FK520 (1), and 7. (A) Fragmentation spectra of 13 (black), 1 (red) and 7 (green). (B) A part of the fragmentation spectra of 13, 1 and 7, which was augmented to show the fragmentation specifics. Differences between 1 and 13 are marked. Light blue markings indicate the presence of the methyl side chain at C21 (found in 13 in 4 (Fig. S7)) and the ethyl side chain at C21 (found in 1 and 7). Light orange markings indicate additional differences between spectra. (C) The FK506 core structure is shown, depicting the position of the key fragment containing position C21 (*m/z* = 223^+^ vs. 237^+^) used for structural confirmation (highlighted light blue).

**
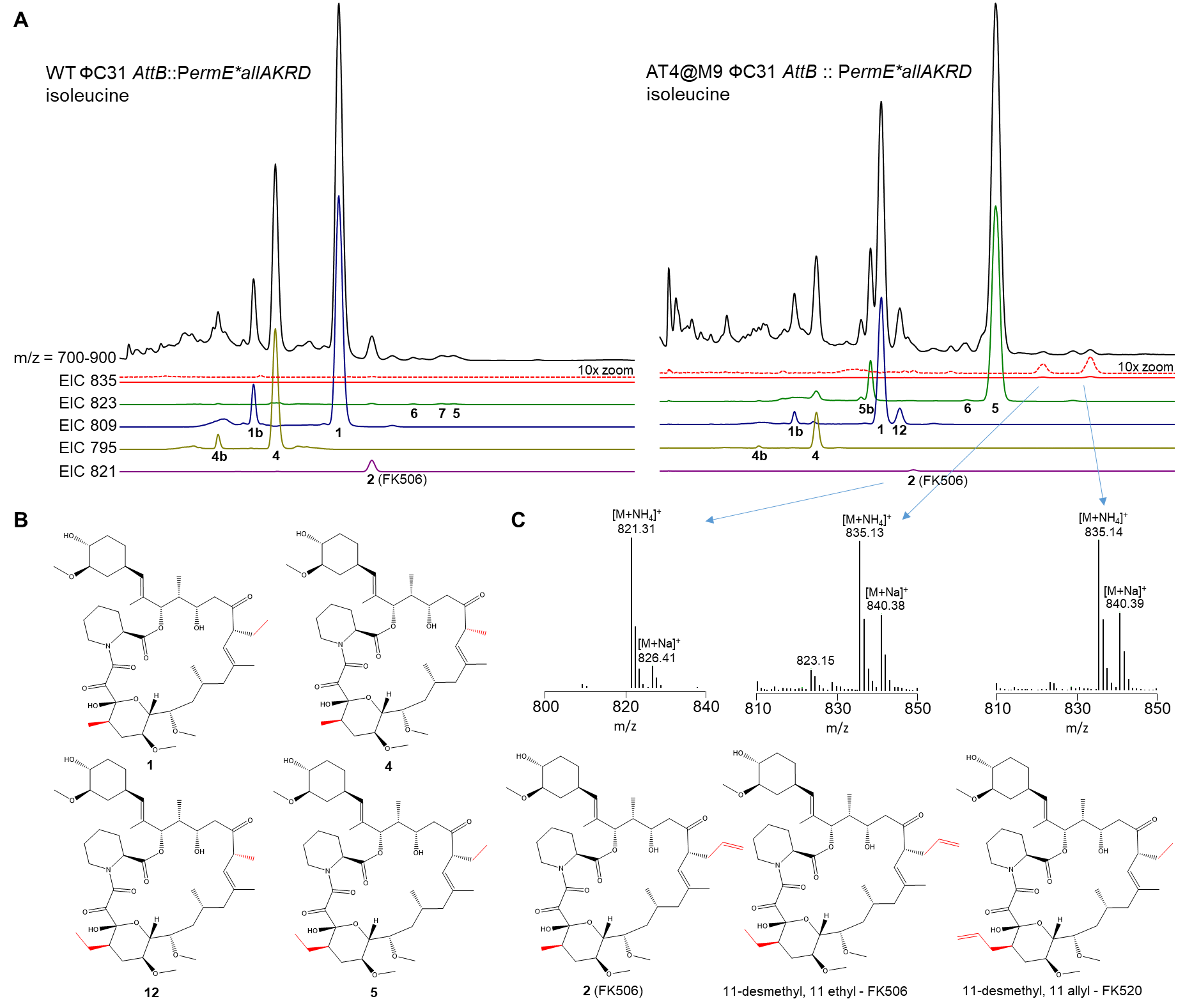
**

Fig. S11 Behavior of the WT and engineered PKS upon introduction of allmal supply (allmal biosynthetic genes *allAKRD* from *S. tsukubaensis* (ref) in addition to the intrinsic mmal and emal supply. WT PKS afforded ~ 5% of FK506 in addition to 1 and 4. With the engineered PKS AT4@AT9, an array of side chain combinations at C11 and C21 was observed. (A) Extracted chromatograms (ESI+, *m/z* = 700-900, 835, 823, 809, 795 and 821) in the time frame of 2-20 min. *S. ascomycinicus* H076 (left panel) and *S. ascomycinicus* AT4@M9 (right panel), both expressing *S. tsukubaensis* *allAKRD* genes from *ermE** promoter and grown on media supplemented with 12 g L^-1^ Ile. All chromatograms are presented on a fixed absolute intensity axis with the added 10× zoom for *m/z* = 835^+^. (B) The proposed structures of all the analogues identified in *S. ascomycinicus* AT4@M9, P*ermE*allAKRD*. Note that it was not determined which of the two C11, C21 regioisomers with ethyl, allyl combinations (*m/z* = 835^+^) is eluting in which of the two peaks. The double: C11, C21 allyl analogue (*m/z* = 847^+^) could not be reliably detected, and is therefore not shown here, although hints of peaks with this mass were found in several samples. (C) MS spectra of the allmal-containing structural analogues.

**Table S3 The composition of the TAA4 medium and the mineral solution used within.** The natural pH is ~ 7.0, there is no pH adjustment prior to sterilization. Sterilization is performed at 121 °C for 20 minutes.

| **Material** | **Amount** |
| --- | --- |
| Corn dextrin (ROQUETTE) | 10 g |
| K_2_HPO_4_ | 1 g |
| MgSO_4_ × 7H_2_O | 1 g |
| NaCl | 1 g |
| (NH_4_)_2_SO_4_ × 7H_2_O | 4 g |
| CaCO_3_ | 4 g |
| Mineral solution | 1 mL |
| Bacto agar (DIFCO BD) | 20 g |
| Tap water | To a final volume of 1 liter |
| Mineral solution: |  |
| **Material** | **Amount** |
| 5 M HCl | 10 mL |
| CaCl_2_ × 2H_2_O | 25 g |
| NaCl | 12 g |
| MgCl_2_ × 6H_2_O | 66 g |
| KCl | 100 g |
| FeCl_3_ × 6H_2_O | 5 g |
| ZnCl_2_ | 0.7 g |
| CuCl_2_ × 2H_2_O | 0.3 g |
| MnSO_4_ × H_2_O | 0.4 g |
| Ultra pure water | To a final volume of 1 liter |

**Table S4** **The composition of the seed culture medium.** The pH is adjusted to 7.2 with 4 M NaOH prior to sterilization. Sterilization is performed at 121 °C for 30 minutes. pH after sterilization was measured to be 6.9 ± 0.1 and was not corrected.

| **Material** | **Amount** |
| --- | --- |
| Glucose | 5 g |
| Soluble starch (Difco BD) | 10 g |
| Corn steep powder (ROQUETTE) | 2.5 g |
| Yeast extract (Difco BD) | 5 g |
| Glycerol | 10 g |
| CaCO_3_ | 1 g |
| Demineralized water | To a final volume of 1 Liter |

**Table S5** **The composition of the main culture medium.** The pH is adjusted to 7.2 with 4 M NaOH prior to sterilization. Sterilization is performed at 121 °C for 30 minutes. pH after sterilization was measured to be 6.7 ± 0.1 and was not corrected. Where indicated, 12 g L^-1^ isoleucine, valine or leucine was added to the medium before sterilization.

| **Material** | **Amount** |
| --- | --- |
| Corn dextrin (ROQUETTE) | 90 g |
| Yeast extract (Difco BD) | 20 g |
| Corn steep powder (ROQUETTE) | 5 g |
| K_2_HPO_4_ | 5 g |
| CaCO_3_ | 0.5 g |
| Demineralized water | To a final volume of 1 Liter |


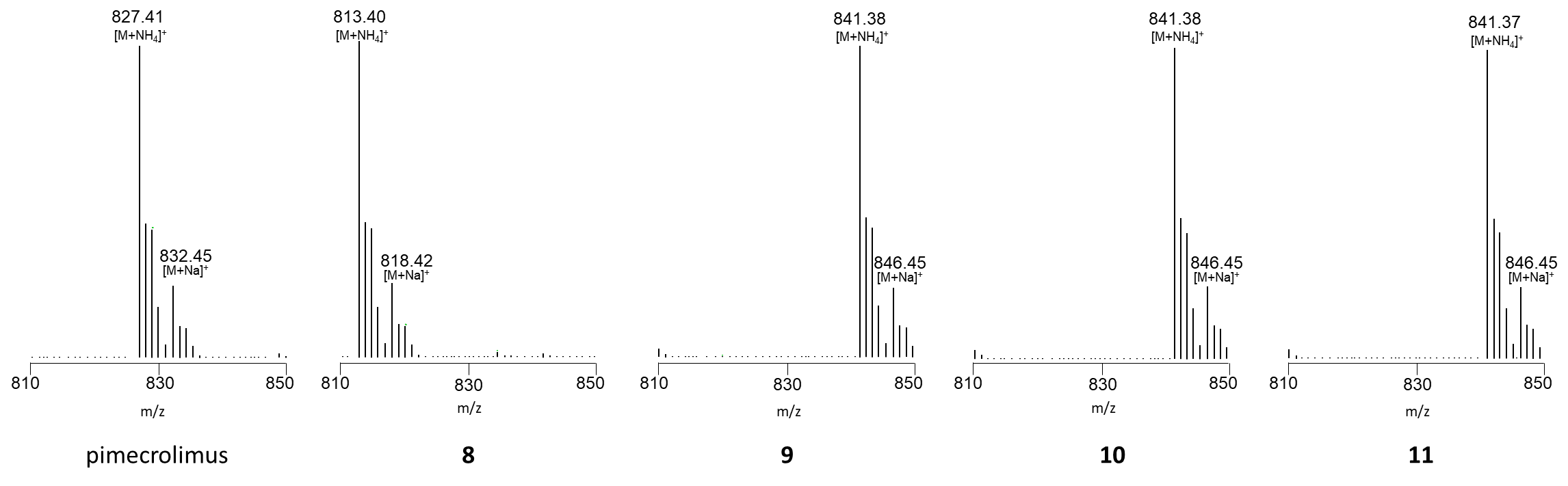


Fig. S12 MS spectra of chlorinated FK520 analogues. Spectra are taken from chromatograms in Fig.4B (main peaks).

**Table S6** **Products of chlorination reactions after quenching.** Reactions were quantified using the UV detector (λ = 210 nm), identity of the products was determined using the MS detector.

| **Substrate** | **Residual substrate** | **Chlorination product** | **Dehydration product** |
| --- | --- | --- | --- |
| **1**  **4** | 15%  7% | 78% (pimecrolimus)  81% (**8**) | 7% (23-24 dehydro-pimecrolimus)  12% (**8**-H_2_O) |
| **5** | 31% | 66% (**9**) | 4% (**9**-H_2_O) |
| **6** | 9% | 82% (**10**) | 9% (**10**-H_2_O) |
| **7** | 16% | 80% (**11**) | 4% (**11**-H_2_O) |
