# Supplementary material for: Engineering of FK520 polyketide synthase for rapid access to quality control reference standards
Source: Microb Cell Fact. 2025 Nov 28;24:243. doi: 10.1186/s12934-025-02861-3 (PMC12664185; doi:10.1186/s12934-025-02861-3)
Supplement: Supplementary file 2 — Supplementary Material 2. [file 12934_2025_2861_MOESM2_ESM.docx]

Supplementary Information 2 for:

**Engineering of FK520 Polyketide Synthase for Rapid Access to Quality Control Reference Standards**

Nina Žigart, Petra Pivk Lukančič, Tjaša Drčar, Jan Peterka, Maja Harej Perko and Peter Mrak*

*peter.mrak@sandoz.com

**This PDF file includes:**

Figs. S11 to S30

Tables S7 to S14

**Table S7**. ^1^H-NMR (600 MHz, CDCl_3_) spectrum annotation for 21-desmethyl-FK520 (**4**)

21-desmethyl-FK520 (**4**)

| **Position** | **Chemical shift (ppm)** | **Number of protons** | **Multiplicity** | **Coupling constant (Hz)** |
| --- | --- | --- | --- | --- |
| CDCl_3_ | 7.26 |  | s |  |
| 26 | 5.36, 5.20 | 1 | 2*m |  |
| 20 | 5.09, 5.07 | 1 | 2*m |  |
| 28 | 5.06, 5.04 | 1 | 2*m |  |
| 2 | 4.62, 4.99 | 1 | 2*m |  |
| 6’ | 4.43, 3.71 | 1 | 2*m |  |
| 10-OH | 4.18, 4.88 | 1 | 2*s |  |
| 24 | 3.89, 3.96 | 1 | 2*m |  |
| 14 | 3.67, 3.86 | 1 | 2*m |  |
| 15 | 3.57 | 1 | 2*m |  |
| 43 | 3.40 | 3 | s |  |
| 13 | 3.39, 3.44 | 1 | 2*m |  |
| 41 | 3.39, 3.37 | 3 | 2*s |  |
| 32 | 3.38 | 1 | m |  |
| 21 | 3.33, 3.34 | 1 | 2*m |  |
| 42 | 3.29, 3.32 | 3 | 2*s |  |
| 6'' | 3.01, 3.28 | 1 | 2*m |  |
| 31 | 3.00 | 1 | m |  |
| 23' | 2.84, 2.76 | 1 | 2*dd | 16.3, 2.2; 17.5, 2.4 |
| 29 | 2.29 | 1 | m |  |
| 11 | 2.15, 2.27 | 1 | 2*m |  |
| 12' | 2.15, 2.09 | 1 | 2*m |  |
| 18' | 2.12, 2.11 | 1 | 2*m |  |
| 3' | 2.09, 2.30 | 1 | 2*m |  |
| 30' | 2.03, 2.02 | 1 | 2*m |  |
| 33' | 1.98 | 1 | 2*m |  |
| 23'' | 1.95, 2.32 | 1 | 2*m |  |
| 3'' | 1.95, 1.77 | 1 | 2*m |  |
| 25 | 1.93, 1.90 | 1 | 2*m |  |
| 18'' | 1.74, 1.90 | 1 | 2*m |  |
| 4' | 1.74, 1.78 | 1 | 2*m |  |
| 5' | 1.74, 1.66 | 1 | 2*m |  |
| 17 | 1.66 | 1 | m |  |
| 40 | 1.65 | 3 | s |  |
| 34' | 1.61 | 1 | 2*m |  |
| 16' | 1.60, 1.56 | 1 | 2*m |  |
| 38 | 1.59, 1.63 | 3 | 2*s |  |
| 12'' | 1.45, 1.51 | 1 | 2*m |  |
| 5'' | 1.43, 1.54 | 1 | 2*m |  |
| 4'' | 1.36, 1.39 | 1 | 2*m |  |
| 33'' | 1.34 | 1 | m |  |
| 16'' | 1.01, 1.32 | 1 | 2*m |  |
| 37 | 1.11, 1.14 | 3 | 2*d | 6.8; 6.9 |
| 34'' | 1.03 | 1 | m |  |
| 35 | 1.00, 0.96 | 3 | 2*d | 6.3; 6.6 |
| 36 | 0.95, 0.83 | 3 | 2*d | 6.2; 6.6 |
| 30'' | 0.92, 0.97 | 1 | 2*m |  |
| 39 | 0.86, 0.91 | 3 | 2*d | 7.3; 7.1 |

**Table S8**. ^13^C-NMR (150 MHz, CDCl_3_) spectrum annotation for 21-desmethyl-FK520 (**4**)

^^

21-desmethyl-FK520 (**4**)

| **Position** | **Chemical shift (ppm)** | **Number of carbons** | **Multiplicity** |
| --- | --- | --- | --- |
| 22 | 214.5, 214.0 | 1 | 2*s |
| 9 | 196.3, 192.6 | 1 | 2*s |
| 1 | 168.9, 168.7 | 1 | 2*s |
| 8 | 164.6, 165.8 | 1 | 2*s |
| 19 | 137.8, 138.6 | 1 | 2*s |
| 27 | 132.8, 131.8 | 1 | 2*s |
| 28 | 129.0, 129.5 | 1 | 2*s |
| 20 | 124.1, 124.5 | 1 | 2*s |
| 10 | 96.8, 98.6 | 1 | 2*s |
| 31 | 84.1 | 1 | s |
| 26 | 75.9., 77.7 | 1 | 2*s |
| CDCl_3_ | 77.0 |  | t |
| 15 | 75.1, 76.5 | 1 | 2*s |
| 13 | 73.6 | 1 | s |
| 32 | 73.54, 73.50 | 1 | 2*s |
| 14 | 72.8, 72.3 | 1 | 2*s |
| 24 | 70.4, 69.1 | 1 | 2*s |
| 42 | 56.8, 57.6 | 1 | 2*s |
| 2 | 56.62, 52.7 | 1 | 2*s |
| 43 | 56.60, 56.55 | 1 | 2*s |
| 41 | 56.3, 56.1 | 1 | 2*s |
| 18 | 48.2, 48.1 | 1 | 2*s |
| 21 | 47.0, 47.2 | 1 | 2*s |
| 23 | 41.5, 42.9 | 1 | 2*s |
| 25 | 39.7, 40.3 | 1 | 2*s |
| 6 | 39.2, 43.9 | 1 | 2*s |
| 30 | 34.89, 34.7 | 1 | 2*s |
| 29 | 34.83, 34.87 | 1 | 2*s |
| 11 | 34.6, 33.7 | 1 | 2*s |
| 12 | 32.7, 32.6 | 1 | 2*s |
| 16 | 32.5, 35.4 | 1 | 2*s |
| 33 | 31.18, 31.16 | 1 | 2*s |
| 34 | 30.64, 30.61 | 1 | 2*s |
| 3 | 27.9, 26.21 | 1 | 2*s |
| 17 | 26.26, 26.19 | 1 | 2*s |
| 5 | 24.6 | 1 | s |
| 4 | 21.3, 20.8 | 1 | 2*s |
| 36 | 20.7, 19.5 | 1 | 2*s |
| 35 | 16.3, 16,0 | 1 | 2*s |
| 38 | 15.72, 15.74 | 1 | 2*s |
| 37 | 15.3, 16.4 | 1 | 2*s |
| 40 | 14.4., 14.2 | 1 | 2*s |
| 39 | 9.5, 9.9 | 1 | 2*s |


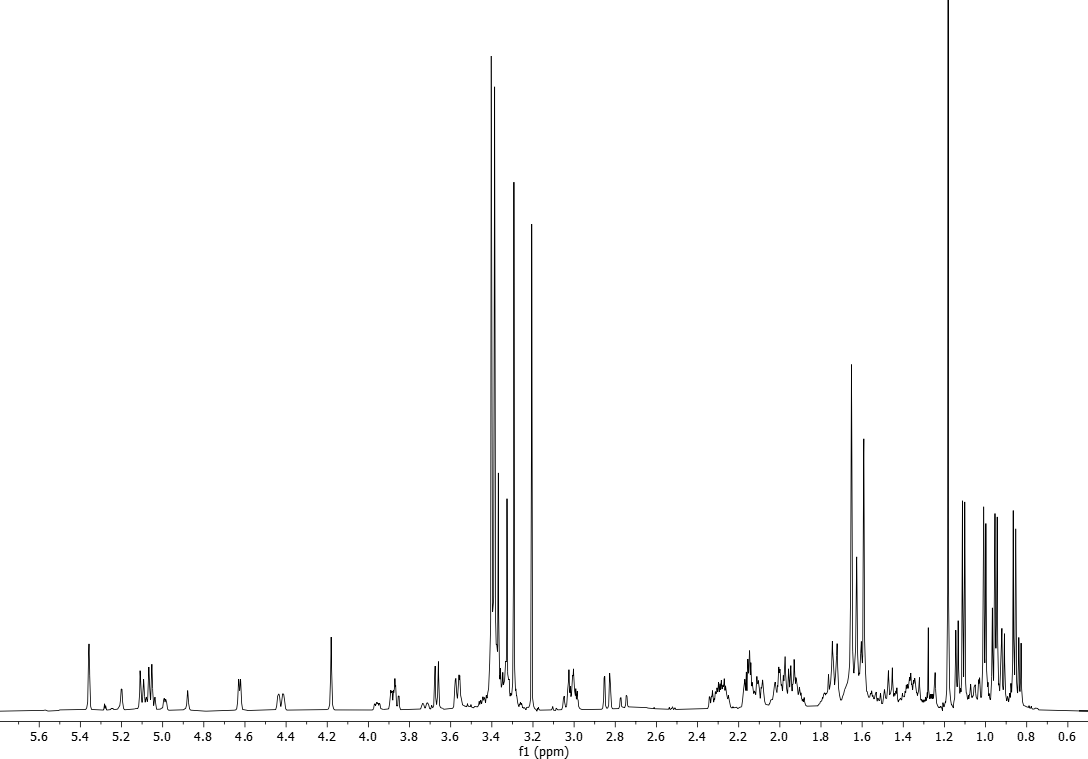


**Fig S11**. ^1^H-NMR (600 MHz, CDCl_3_) spectrum for 21-desmethyl-FK520 (**4**).


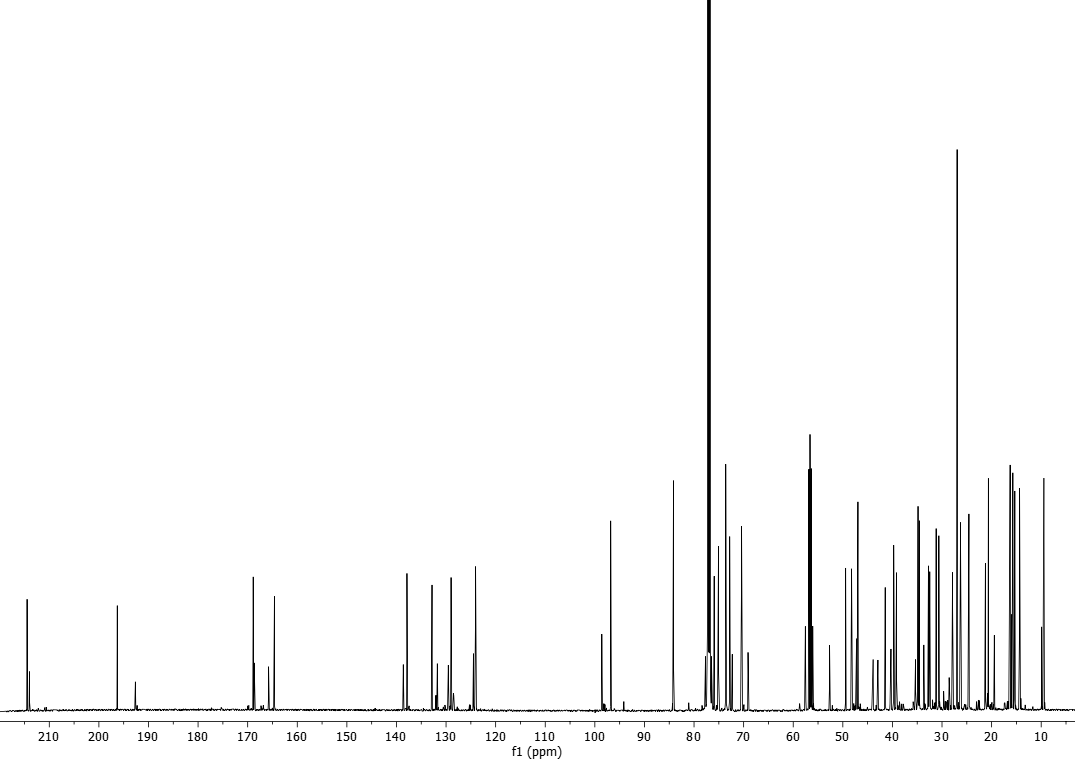


**Fig S12**. ^13^C-NMR (150 MHz, CDCl_3_) spectrum for 21-desmethyl-FK520 (**4**)


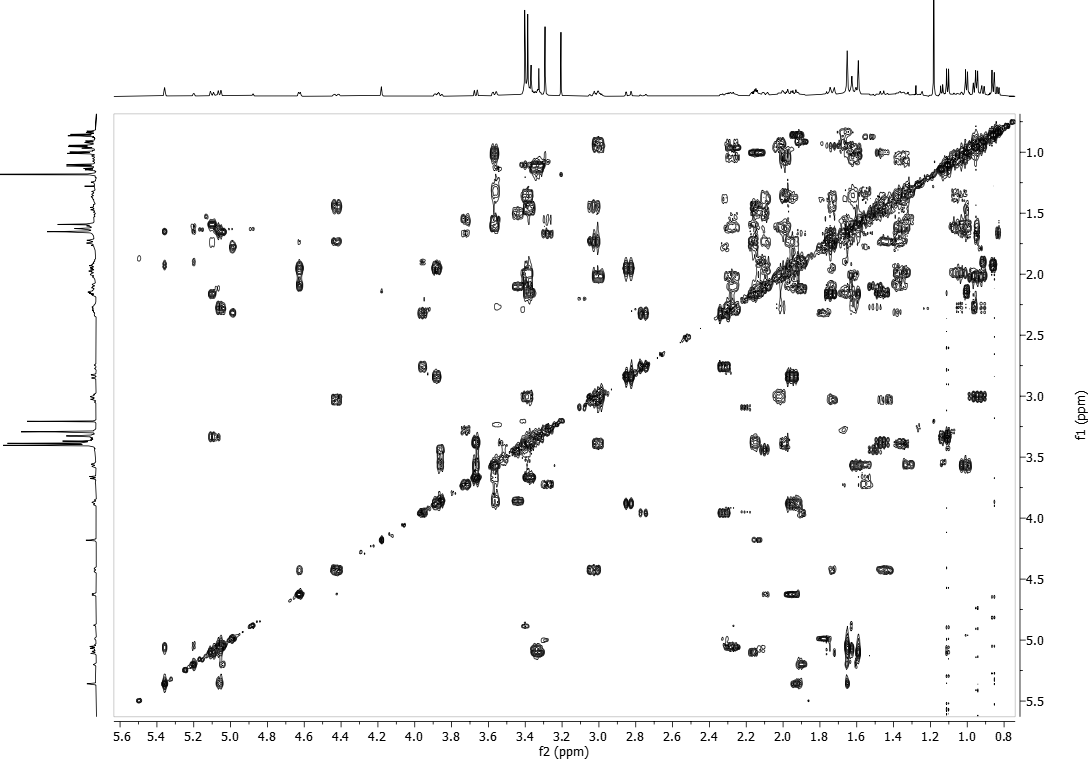


**Fig S13**. COSY 2D spectrum for 21-desmethyl-FK520 (**4**).


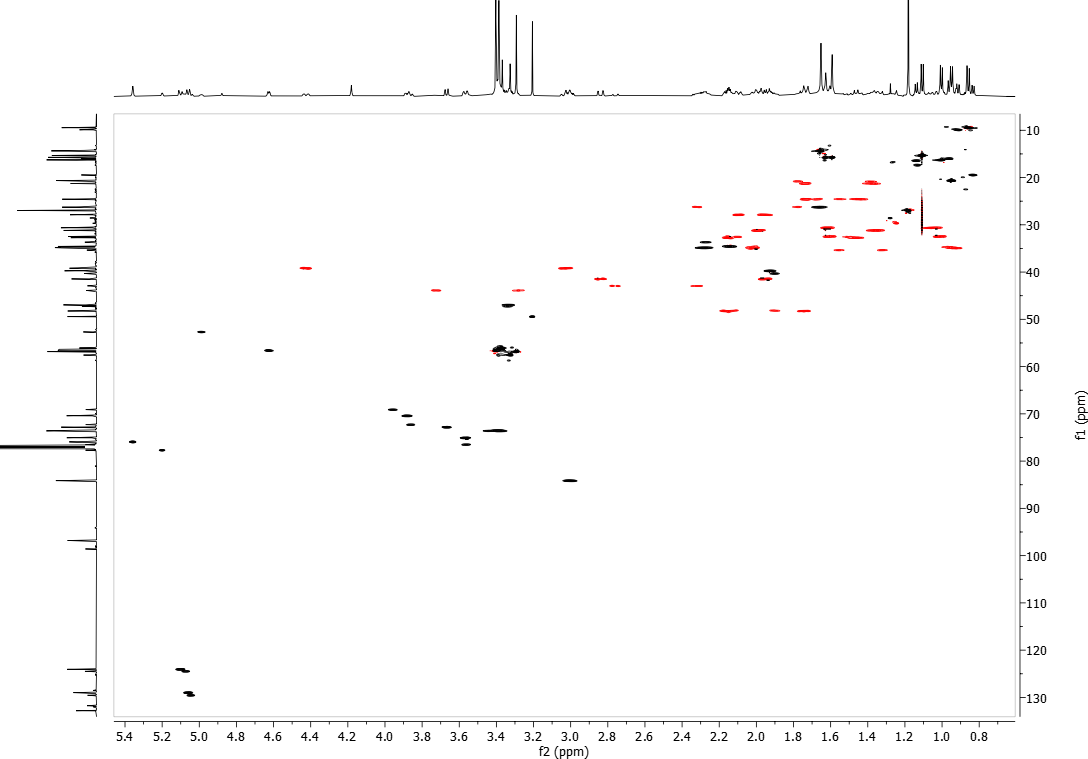


**Fig S14**. HSQC 2D spectrum for 21-desmethyl-FK520 (**4**).


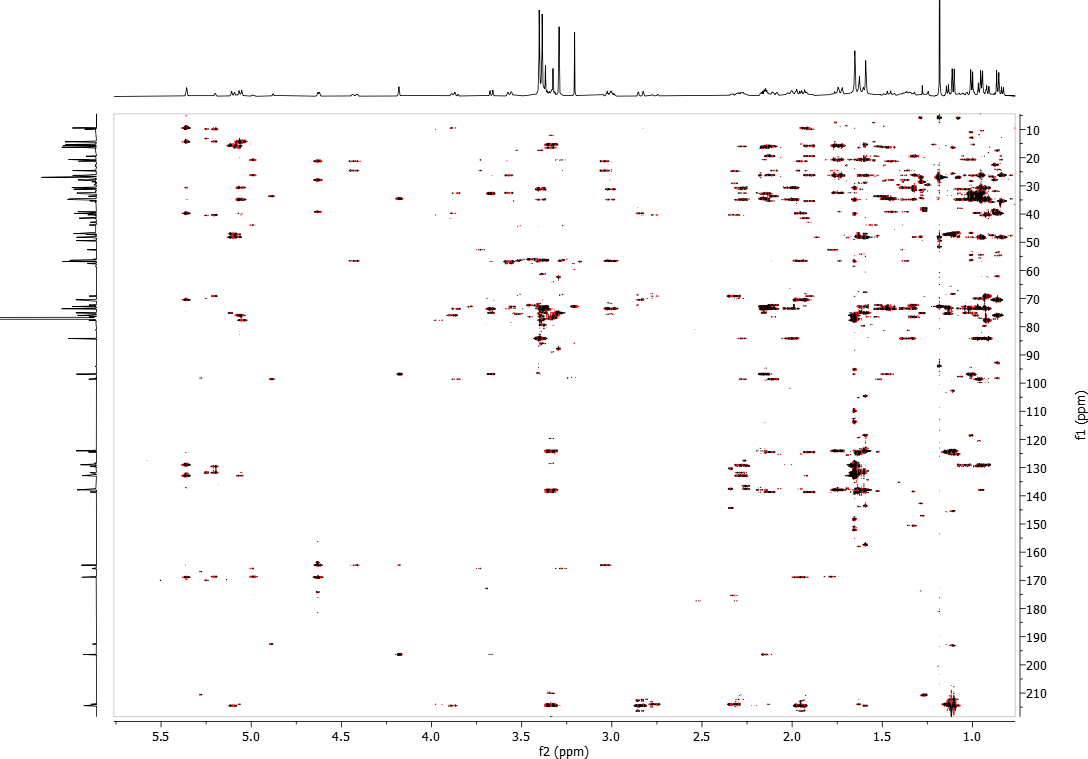
 **Fig S15**. HMBC 2D spectrum for 21-desmethyl-FK520 (**4**)

**Table S9**. ^1^H-NMR (600 MHz, CDCl_3_) spectrum annotation for 11-ethyl-11-desmethyl-Fk520 (**5**)

11-ethyl-11-desmethyl-Fk520 (**5**)

| **Position** | **Chemical shift (ppm)** | **Number of protons** | **Multiplicity** | **Coupling constant (Hz)** |
| --- | --- | --- | --- | --- |
| CDCl_3_ | 7.26 |  | s |  |
| 26 | 5.35, 5.19 | 1 | m |  |
| 28 | 5.08, 5.05 | 1 | d | 9.0; 9.1 |
| 20 | 5.02, 5.00 | 1 | m |  |
| 2 | 4.57, 5.00 | 1 | m |  |
| 10-OH | 4.23, 4.72 | 1 | s |  |
| 6’ | 4.42, 3.72 | 1 | m |  |
| 24 | 3.90, 3.94 | 1 | m |  |
| 14 | 3.67, 3.87 | 1 | m |  |
| 15 | 3.56 | 1 | m |  |
| 13 | 3.37, 3.43 | 1 | m |  |
| 45 | 3.40 | 3 | s |  |
| 43 | 3.40, 3.39 | 3 | 2*s |  |
| 32 | 3.39 | 1 | m |  |
| 44 | 3.29, 3.33 | 3 | 2*s |  |
| 6'' | 3.02, 3.27 | 2 | m |  |
| 21 | 3.21, 3.17 | 1 | m |  |
| 31 | 3.00 | 1 | m |  |
| 23' | 2.79, 2.72 | 1 | 2*dd | 15.8, 2.9;  17.4, 2.8 |
| 12' | 2.38, 2.36 | 1 | 2*m |  |
| 29 | 2.29 | 1 | m |  |
| 18' | 2.15, 2.17 | 1 | 2*m |  |
| 3' | 2.08, 2.31 | 1 | 2*m |  |
| 23'' | 2.05, 2.33 | 1 | 2*m |  |
| 30' | 2.03, 2.02 | 1 | 2*m |  |
| 33' | 2.00 | 1 | m |  |
| 11 | 1.94, 2.06 | 1 | 2*m |  |
| 3'' | 1.94, 1.78 | 1 | 2*m |  |
| 25 | 1.89 | 1 | m |  |
| 18'' | 1.81, 1.91 | 1 | 2*m |  |
| 39' | 1.76, 1.69 | 1 | 2*m |  |
| 4' | 1.74, 1.79 | 1 | 2*m |  |
| 5' | 1.73, 1.68 | 1 | 2*m |  |
| 17 | 1.66, 1.64 | 1 | 2*m |  |
| 42 | 1.62, 1.65 | 3 | 2*s |  |
| 34' | 1.62 | 1 | m |  |
| 38 | 1.59, 1.61 | 3 | 2*s |  |
| 35' | 1.57, 1.47 | 1 | 2*m |  |
| 16' | 1.53, 1.56 | 1 | 2*m |  |
| 5'' | 1.47, 1.54 | 1 | 2*m |  |
| 39'' | 1.43, 1.55 | 1 | 2*m |  |
| 4'' | 1.39, 1.41 | 1 | 2*m |  |
| 33'' | 1.35 | 1 | m |  |
| 12'' | 1.30, 1.34 | 1 | 2*m |  |
| 35'' | 1.16, 1.18 | 1 | 2*m |  |
| 34'' | 1.07 | 1 | m |  |
| 16'' | 1.05, 1.33 | 1 | 2*m |  |
| 30'' | 0.93, 0.98 | 1 | 2*m |  |
| 37 | 0.93, 0.83 | 3 | 2*d | 6.4 |
| 36 | 0.90 | 3 | d | 7.6 |
| 41 | 0.87, 0.91 | 3 | 2*m |  |
| 40 | 0.85 | 3 | m |  |

**Table S10**. ^13^C-NMR (150 MHz, CDCl_3_) spectrum annotation for 11-ethyl-11-desmethyl-Fk520 (**5**)

| **Position** | **Chemical shift (ppm)** | **Number of carbons** | **Multiplicity** |
| --- | --- | --- | --- |
| 22 | 213.5, 213.4 | 1 | 2*s |
| 9 | 196.4, 192.8 | 1 | 2*s |
| 1 | 169.0, 168.8 | 1 | 2*s |
| 8 | 164.6, 165.7 | 1 | 2*s |
| 19 | 138.8, 139.6 | 1 | 2*s |
| 27 | 132.3, 131.8 | 1 | 2*s |
| 28 | 129.7, 129.6 | 1 | 2*s |
| 20 | 123.0, 123.3 | 1 | 2*s |
| 10 | 97.2, 98.7 | 1 | 2*s |
| 31 | 84.1 | 1 | s |
| 26 | 77.2, 77.9 | 1 | 2*s |
| CDCl_3_ | 77.0 |  | t |
| 15 | 75.3, 76.7 | 1 | 2*s |
| 13 | 73.84, 73.79 | 1 | 2*s |
| 32 | 73.53, 73.50 | 1 | 2*s |
| 14 | 73.2, 72.4 | 1 | 2*s |
| 24 | 70.0, 69.0 | 1 | 2*s |
| 44 | 56.9, 57.5 | 1 | 2*s |
| 2 | 56.59, 52.7 | 1 | 2*s |
| 45 | 56.57, 56.55 | 1 | 2*s |
| 43 | 56.4, 56.1 | 1 | 2*s |
| 21 | 54.7, 54.9 | 1 | 2*s |
| 18 | 48.6, 48.4 | 1 | 2*s |
| 23 | 43.1, 43,6 | 1 | 2*s |
| 11 | 41.0, 40.2 | 1 | 2*s |
| 25 | 39.7, 40.3 | 1 | 2*s |
| 6 | 39.2, 43.9 | 1 | 2*s |
| 29 | 34.84, 34.88 | 1 | 2*s |
| 30 | 34.81, 33.7 | 1 | 2*s |
| 16 | 33.0, 35.3 | 1 | 2*s |
| 33 | 32.17, 31.15 | 1 | 2*s |
| 34 | 30.6 | 1 | s |
| 12 | 29.1, 28.9 | 1 | 2*s |
| 3 | 27.5, 26.2 | 1 | 2*s |
| 17 | 26.3, 26.1 | 1 | 2*s |
| 5 | 24.47, 24.60 | 1 | 2*s |
| 39 | 24.2, 24.54 | 1 | 2*s |
| 35 | 23.5, 23.1 | 1 | 2*s |
| 4 | 21.1, 20.9 | 1 | 2*s |
| 37 | 20.5, 19.6 | 1 | 2*s |
| 38 | 15.9, 15.7 | 1 | 2*s |
| 42 | 14.1, 14.2 | 1 | 2*s |
| 40 | 11.7 | 1 | s |
| 36 | 11.3, 11.5 | 1 | 2*s |
| 41 | 9.5, 9.8 | 1 | 2*s |

11-ethyl-11-desmethyl-Fk520 (**5**)


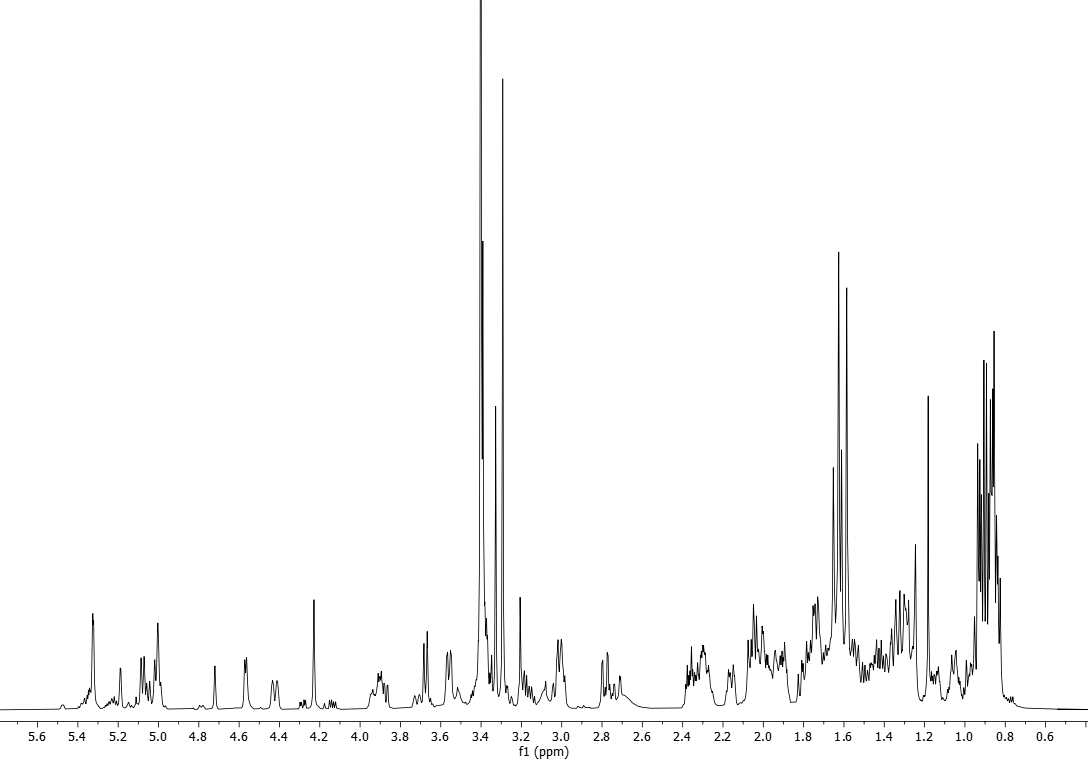


**Fig S16**. ^1^H-NMR (600 MHz, CDCl_3_) spectrum for 11-ethyl-11-desmethyl-Fk520 (**5**).


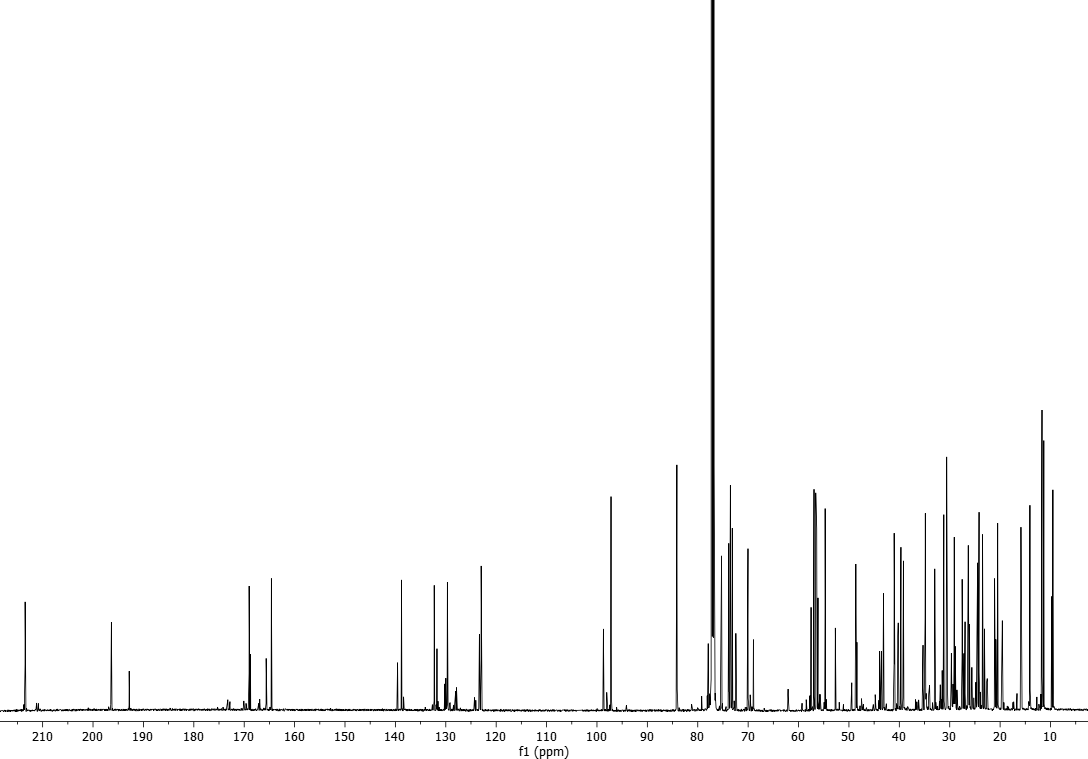


**Fig S17**. ^13^C-NMR (150 MHz, CDCl_3_) spectrum for 11-ethyl-11-desmethyl-Fk520 (**5**).


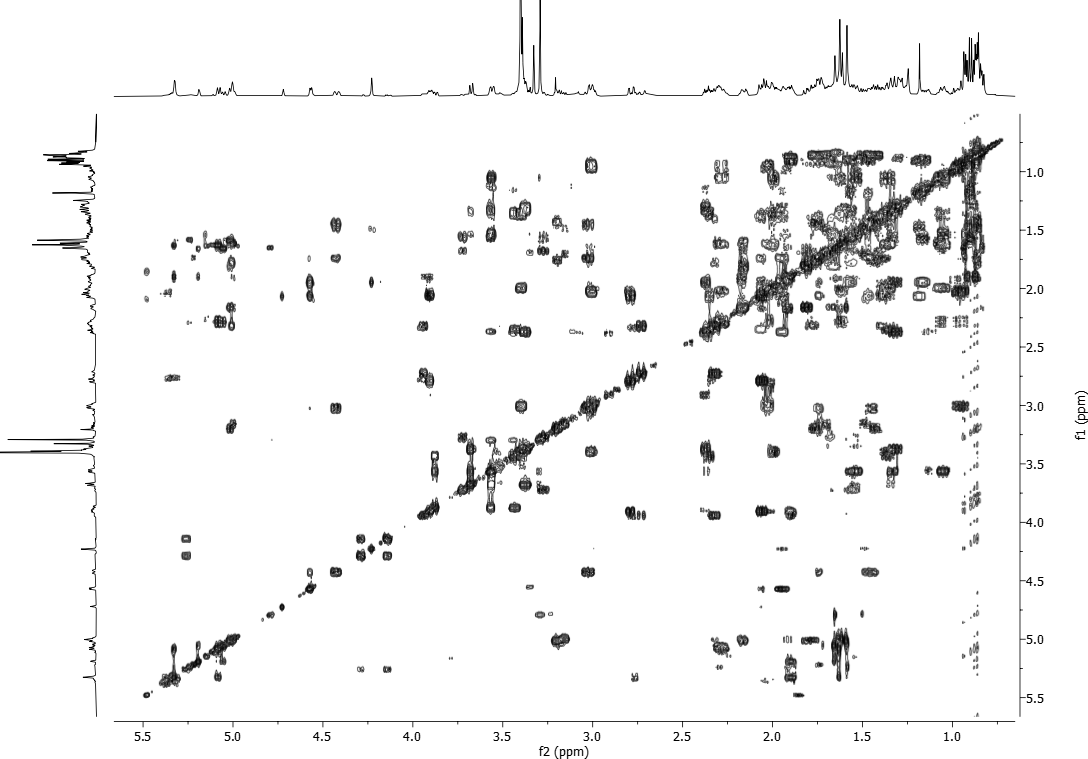


**Fig S18**. COSY 2D spectrum for 11-ethyl-11-desmethyl-Fk520 (**5**).


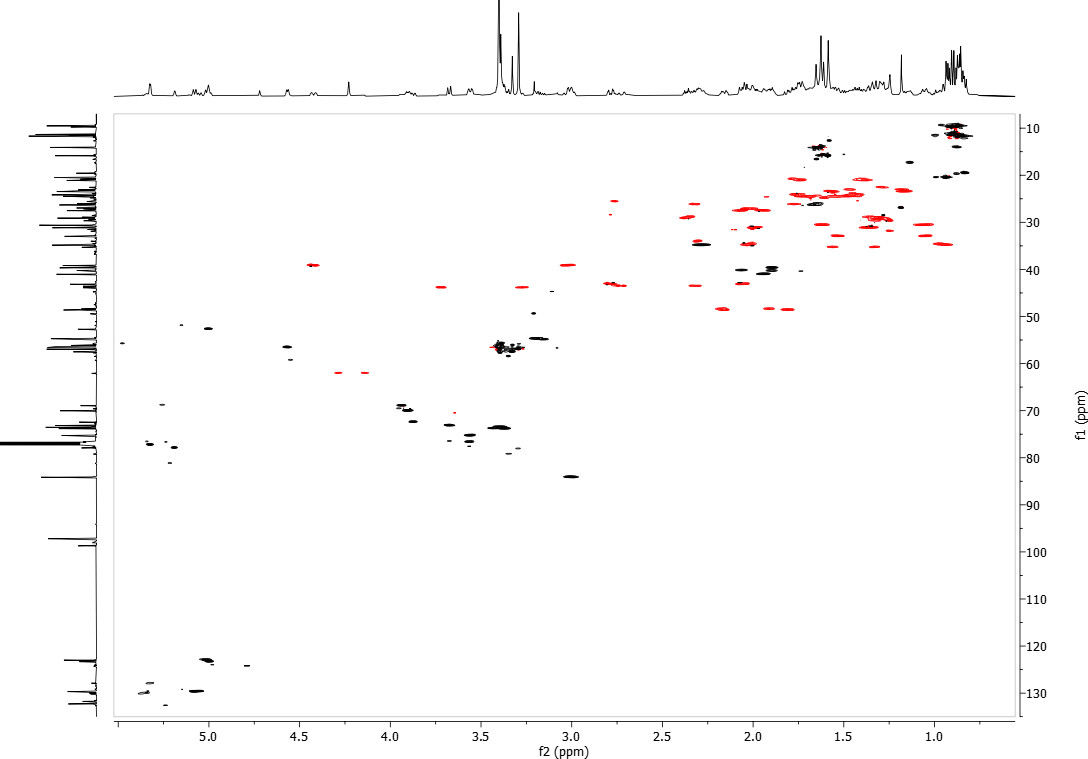


**Fig S19**. HSQC 2D spectrum for 11-ethyl-11-desmethyl-Fk520 (**5**).


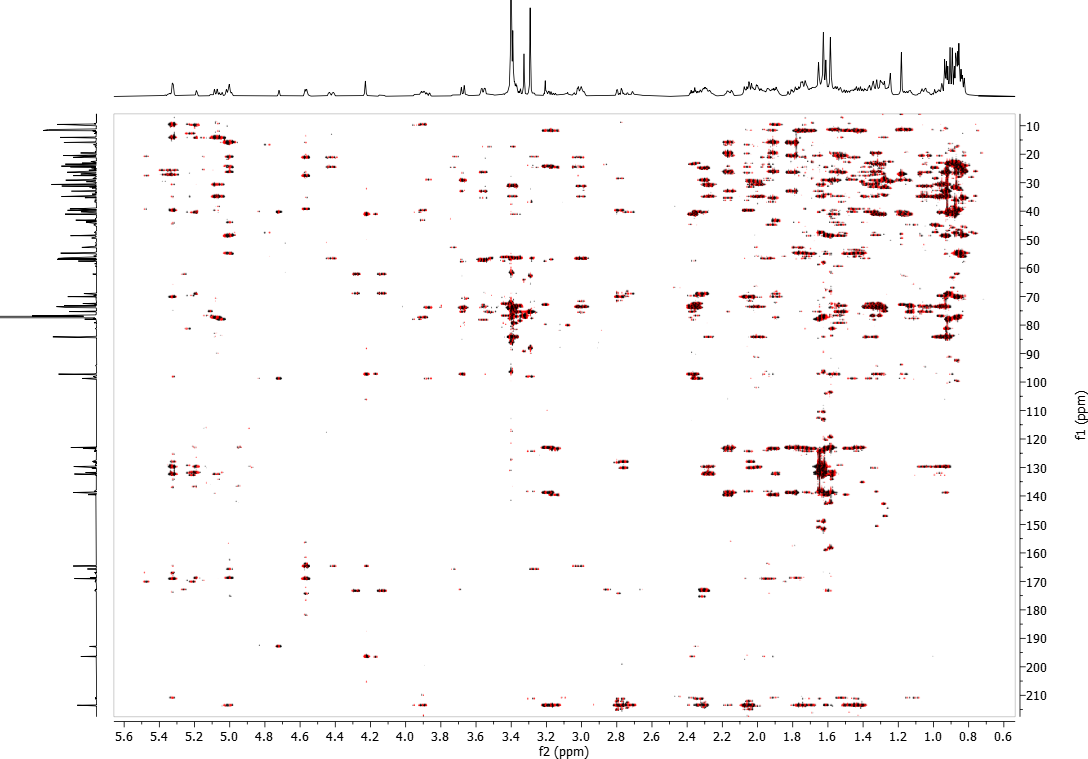


**Fig S20**. HMBC 2D spectrum for 11-ethyl-11-desmethyl-Fk520 (**5**).

**Table S11**. ^1^H-NMR (600 MHz, CDCl_3_) spectrum annotation for 17-ethyl-17-desmethyl-FK520 (**6**).

17-ethyl-17-desmethyl-Fk520 (**5**)

| **Position** | **Chemical shift (ppm)** | **Number of protons** | **Multiplicity** | **Coupling constant (Hz)** |
| --- | --- | --- | --- | --- |
| CDCl_3_ | 7.26 |  |  |  |
| 26 | 5.53, 5.19 | 1 | 2*d | 2.9 |
| 28 | 5.07 | 1 | m |  |
| 20 | 5.03, 4.99 | 1 | 2*m |  |
| 2 | 4.58, 4.98 | 1 | d, m | 5.6 |
| 6’ | 4.42, 3.71 | 1 | 2*m |  |
| 10-OH | 4.28, 4.80 | 1 | 2*s |  |
| 24 | 3.90, 3.94 | 1 | 2*m |  |
| 14 | 3.65, 6.86 | 1 | m |  |
| 15 | 3.53 | 1 | 2*m |  |
| 13 | 3.37, 3.45 | 1 | 2*m |  |
| 45 | 3.40 | 3 | s |  |
| 32 | 3.39 | 1 | m |  |
| 43 | 3.38, 3.37 | 3 | 2*s |  |
| 44 | 3.29,.3.34 | 3 | 2*s |  |
| 21 | 3.20, 3.15 | 1 | 2*m |  |
| 6'' | 3.05, 3.26 | 1 | 2*m |  |
| 31 | 3.01 | 1 | m |  |
| 23' | 2.79, 2.74 | 1 | 2*dd | 15.8, 3.0; 17.6, 2.7 |
| 29 | 2.28 | 1 | m |  |
| 18' | 2.27, 2.16 | 1 | 2*m |  |
| 11 | 2.18, 2.30 | 1 | 2*m |  |
| 12' | 2.14, 2.09 | 1 | 2*m |  |
| 3' | 2.08, 2.31 | 1 | 2*m |  |
| 23'' | 2.05, 2.34 | 1 | 2*m |  |
| 30' | 2.03 | 1 | m |  |
| 33' | 1.98 | 1 | m |  |
| 3'' | 1.94, 1.78 | 1 | 2*m |  |
| 25 | 1.91, 1.89 | 1 | 2*m |  |
| 39' | 1.76, 1.69 | 1 | 2*m |  |
| 18'' | 1.75, 2.02 | 1 | 2*m |  |
| 4' | 1.74, 1.78 | 1 | 2*m |  |
| 5' | 1.74, 1.73 | 1 | 2*m |  |
| 42 | 1.63, 1.65 | 3 | 2*s |  |
| 34' | 1.61, 1.46 | 1 | 2*m |  |
| 38 | 1.60, 1.62 | 3 | 2*s |  |
| 16' | 1.54 | 1 | m |  |
| 17 | 1.51, 1.49 | 1 | 2*m |  |
| 36' | 1.50, 1.29 | 1 | 2*m |  |
| 12'' | 1.48, 1.53 | 1 | 2*m |  |
| 5'' | 1.47, 1.54 | 1 | 2*m |  |
| 16'' | 1.45 | 1 | m |  |
| 39'' | 1.43, 1.47 | 1 | 2*m |  |
| 4'' | 1.38 | 1 | m |  |
| 33'' | 1.35 | 1 | m |  |
| 36'' | 1.20 | 1 | m |  |
| 34'' | 1.04, 1.19 | 1 | 2*m |  |
| 35 | 0.98, 0.94 | 3 | 2*d | 6.4; 6.7 |
| 30'' | 0.95, 0.97 | 1 | 2*m |  |
| 37 | 0.90, 0.87 | 3 | d, m | 5.3 |
| 41 | 0.86, 0.91 | 3 | m, d | 5.1 |
| 40 | 0.86 | 3 | m |  |

**Table S12**. ^13^C-NMR (150 MHz, CDCl_3_) spectrum annotation for 17-ethyl-17-desmethyl-FK520 (**6**).

17-ethyl-17-desmethyl-Fk520 (**6**)

| Position | Chemical shift (ppm) | Number of carbons | Multiplicity |
| --- | --- | --- | --- |
| 22 | 213.6, 213.5 | 1 | 2*s |
| 9 | 196.1, 192.8 | 1 | 2*s |
| 1 | 169.0, 168.7 | 1 | 2*s |
| 8 | 164.7, 165.9 | 1 | 2*s |
| 19 | 139.0, 139.8 | 1 | 2*s |
| 27 | 132.3, 131.7 | 1 | 2*s |
| 28 | 129.7, 129.6 | 1 | 2*s |
| 20 | 123.1, 123.5 | 1 | 2*s |
| 10 | 97.1, 98.7 | 1 | 2*s |
| 31 | 84.1 | 1 | s |
| 26 | 77.2, 77.9 | 1 | 2*s |
| CDCl_3_ | 77.0 |  | t |
| 15 | 75.1, 77.2 | 1 | 2*s |
| 13 | 73.8 | 1 | s |
| 32 | 73.53, 73.50 | 1 | 2*s |
| 14 | 73.0, 72.5 | 1 | 2*s |
| 24 | 70.0, 69.0 | 1 | 2*s |
| 44 | 57.0, 57.7 | 1 | 2*s |
| 2 | 56.59, 52.7 | 1 | 2*s |
| 45 | 56.59, 56.55 | 1 | 2*s |
| 43 | 56.3, 56.0 | 1 | 2*s |
| 21 | 54.7, 54.8 | 1 | 2*s |
| 18 | 44.8, 45.9 | 1 | 2*s |
| 23 | 43.0, 43,6 | 1 | 2*s |
| 25 | 39.7, 40.1 | 1 | 2*s |
| 6 | 39.2, 43.9 | 1 | 2*s |
| 29 | 34.84, 34.9 | 1 | 2*s |
| 30 | 34.82, 34.7 | 1 | 2*s |
| 11 | 34.6, 33.7 | 1 | 2*s |
| 12 | 32.6, 32.3 | 1 | 2*s |
| 16 | 32.14, 32.08 | 1 | 2*s |
| 17 | 31.6 | 1 | s |
| 33 | 31.16, 31.17 | 1 | 2*s |
| 34 | 30.6, 30.4 | 1 | 2*s |
| 3 | 27.6, 26.1 | 1 | 2*s |
| 36 | 26.3, 26.0 | 1 | 2*s |
| 5 | 24.46, 24.53 | 1 | 2*s |
| 39 | 24.2, 24.6 | 1 | 2*s |
| 4 | 21.1, 20.8 | 1 | 2*s |
| 35 | 16.2, 16.0 | 1 | 2*s |
| 38 | 15.9, 15.7 | 1 | 2*s |
| 42 | 14.1, 14.2 | 1 | 2*s |
| 40 | 11.66, 11.69 | 1 | 2*s |
| 37 | 10.5, 10.4 | 1 | 2*s |
| 41 | 9.5, 9.8 | 1 | 2*s |


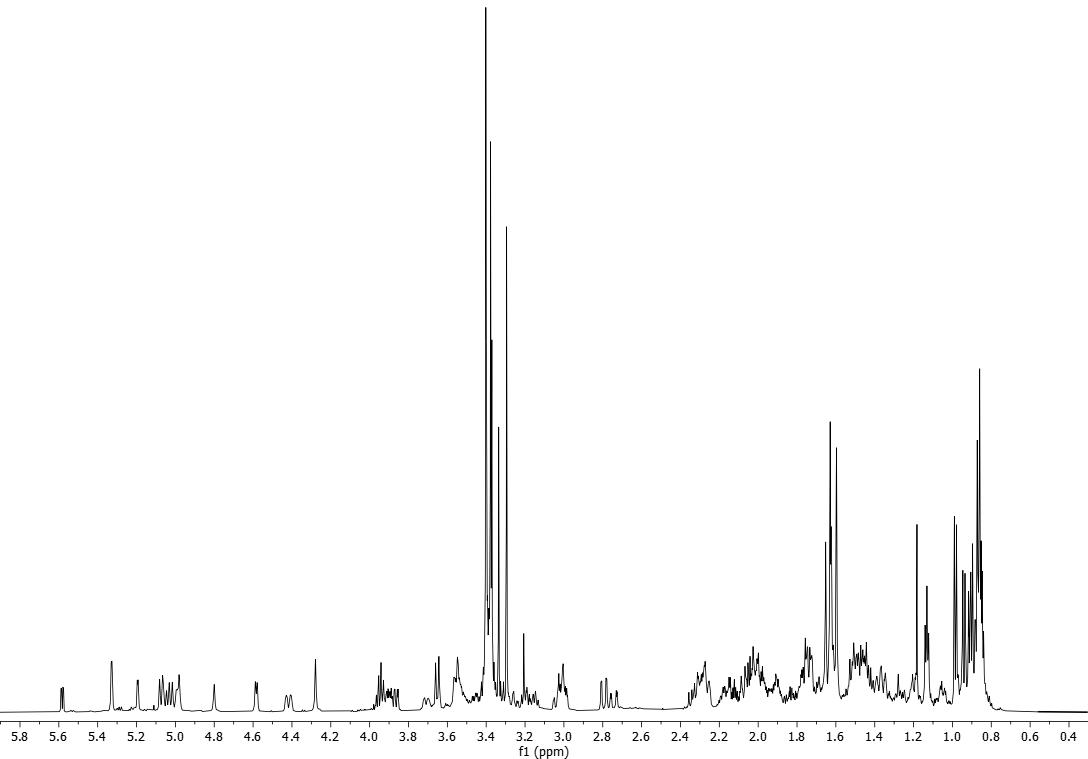


**Fig S21**. ^1^H-NMR (600 MHz, CDCl_3_) spectrum for 17-ethyl-17-desmethyl-FK520 (**6**).


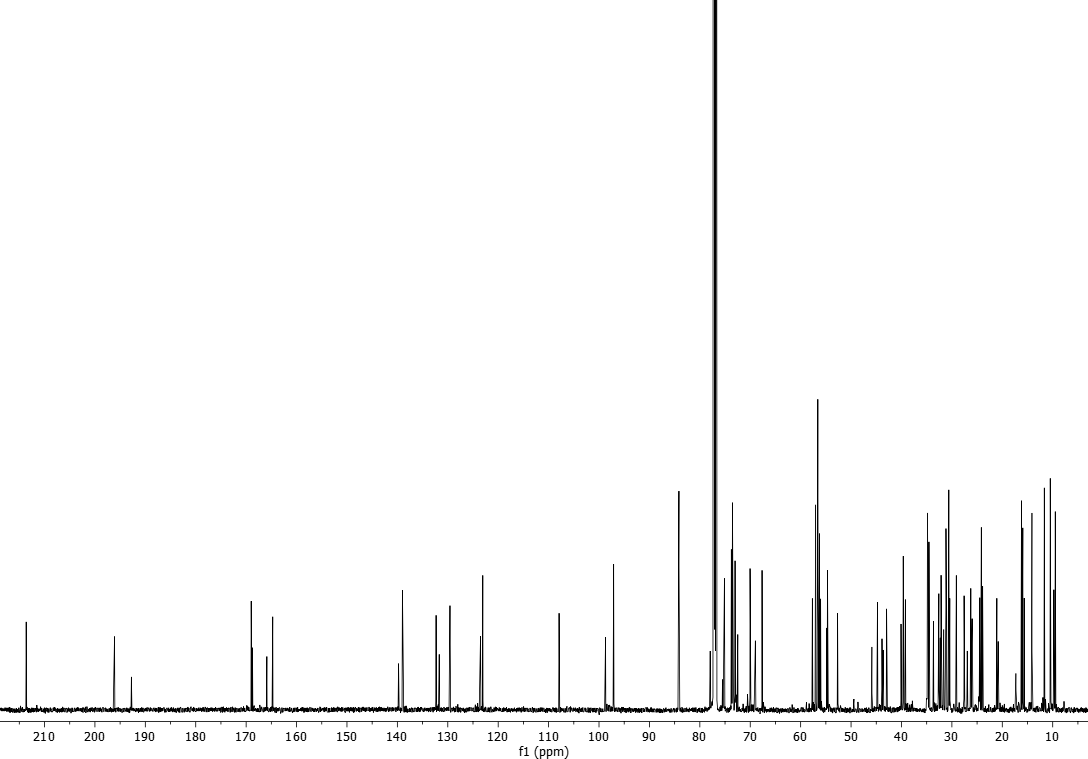


**Fig S22**. ^13^C-NMR (150 MHz, CDCl_3_) spectrum for 17-ethyl-17-desmethyl-FK520 (**6**).


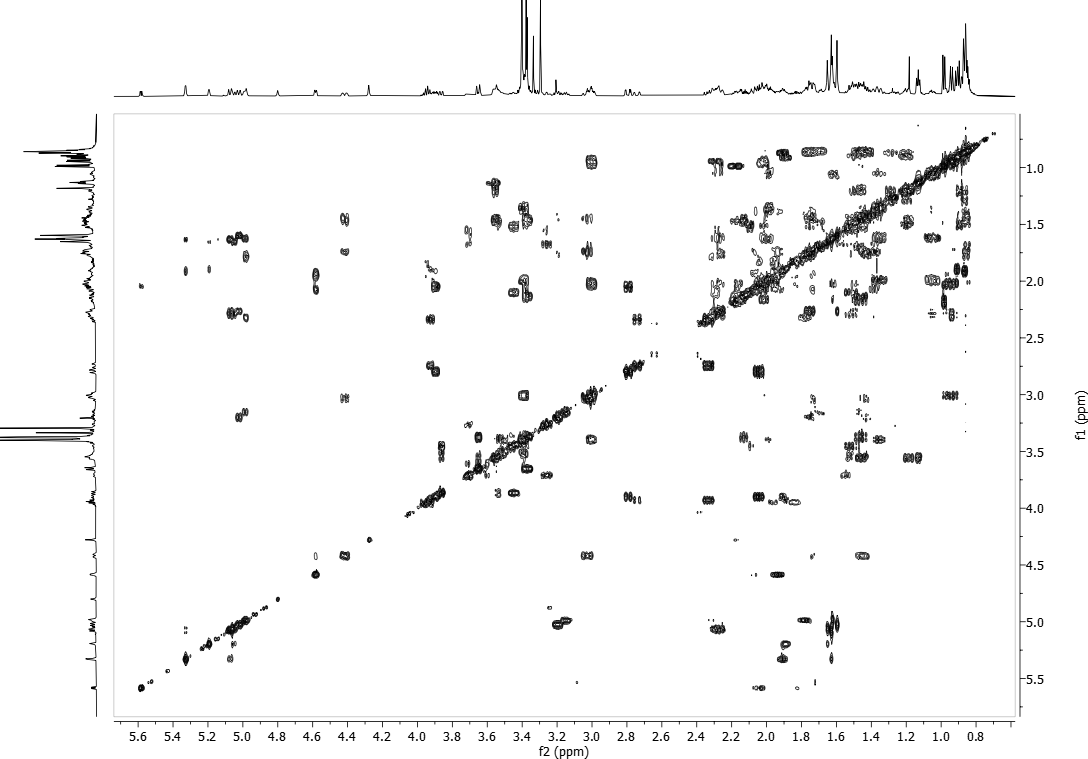


**Fig S23**. COSY 2D spectrum for 17-ethyl-17-desmethyl-FK520 (**6**).


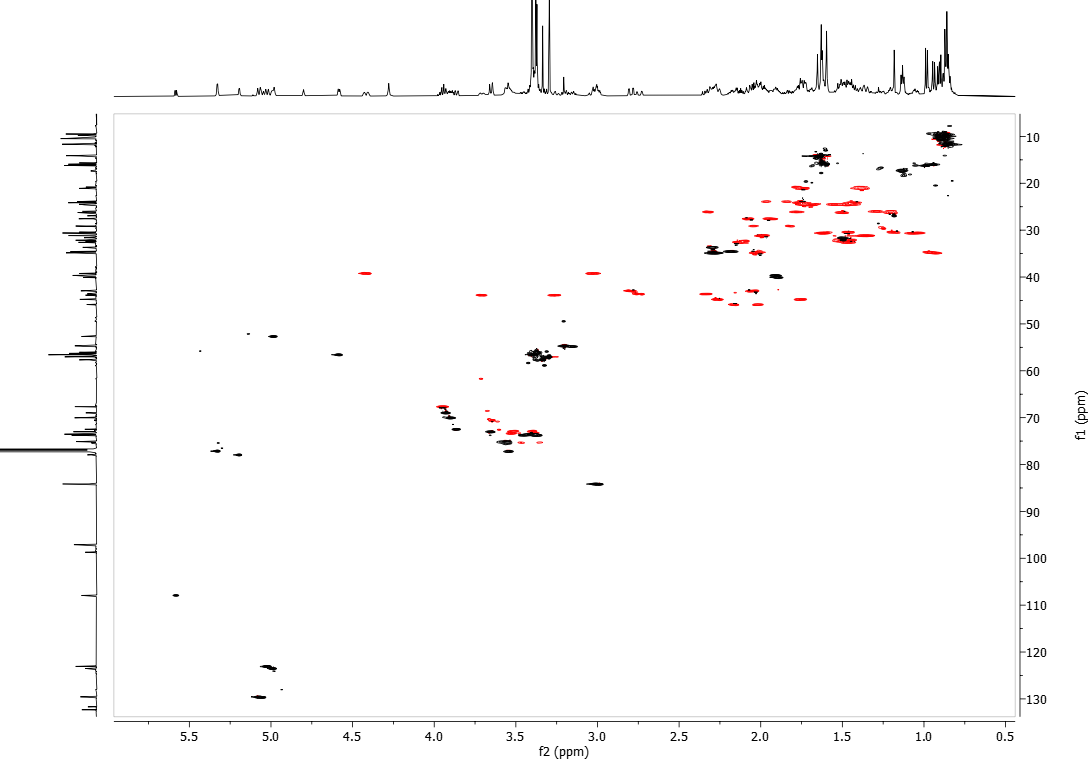


**Fig S24**. HSQC 2D spectrum for 17-ethyl-17-desmethyl-FK520 (**6**).


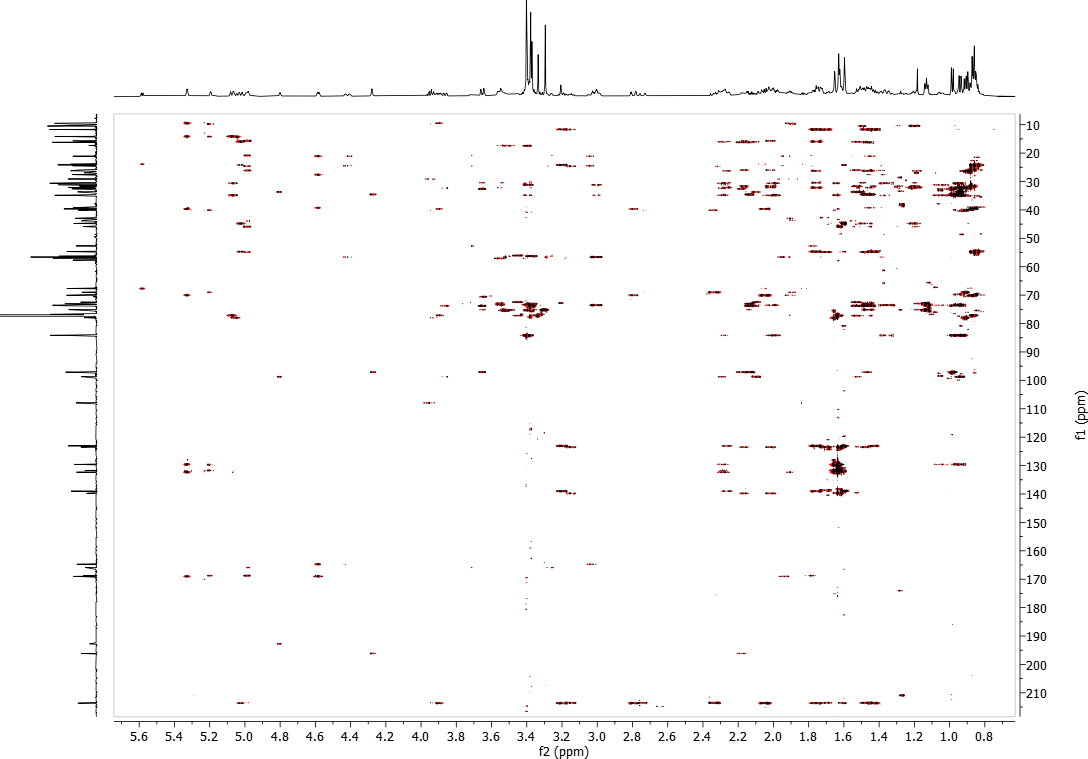


**Fig S25**. HMBC 2D spectrum for 17-ethyl-17-desmethyl-FK520 (**6**).

**Table S13**. ^1^H-NMR (600 MHz, CDCl_3_) spectrum annotation for 19-ethyl-19-desmethyl-FK520 (**7**).

19-ethyl-19-desmethyl-Fk520 (**7**)

| **Position** | **Chemical shift (ppm)** | **Number of protons** | **Multiplicity** | **Coupling constant (Hz)** |
| --- | --- | --- | --- | --- |
| CDCl_3_ | 7.26 |  |  |  |
| 26 | 5.32, 5.21 | 1 | 2*m |  |
| 28 | 5.06 | 1 | m |  |
| 20 | 4.99, 5.05 | 1 | d, m | 9.8 |
| 2 | 4.61, 5.03 | 1 | 2*m |  |
| 10-OH | 4.22, 4.62 | 1 | 2*s |  |
| 6’ | 4.43, 3.63 | 1 | 2*m |  |
| 24 | 3.90, 3.95 | 1 | 2*m |  |
| 14 | 3.66, 3.83 | 1 | m, dd | 9.5, 2.7 |
| 15 | 3.55 | 1 | m |  |
| 13 | 3.36, 3.43 | 1 | 2*m |  |
| 32 | 3.39 | 1 | m |  |
| 45 | 3.40, 3.38 | 3 | 2*s |  |
| 43 | 3.38, 3.37 | 3 | 2*s |  |
| 44 | 3.29, 3.32 | 3 | 2*s |  |
| 21 | 3.14, 3.22 | 1 | 2*m |  |
| 6'' | 2.92, 3.30 | 1 | dd, m | 13.1, 2.8 |
| 31 | 3.01 | 1 | m |  |
| 23' | 2.75, 2.76 | 1 | 2*dd | 16.0, 2.6; 17.5, 3.3 |
| 18' | 2.33, 2.36 | 1 | 2*m |  |
| 29 | 2.28, 2.29 | 1 | 2*m |  |
| 37' | 2.16, 2.04 | 1 | 2*m |  |
| 12' | 2.15, 2.10 | 1 | 2*m |  |
| 11 | 2.14, 2.34 | 1 | 2*m |  |
| 23'' | 2.08, 2.37 | 1 | 2*m |  |
| 3' | 2.06, 2.30 | 1 | 2*m |  |
| 30' | 2.01, 2.03 | 1 | 2*m |  |
| 33' | 1.98 | 1 | m |  |
| 3'' | 1.95, 1.78 | 1 | 2*m |  |
| 25 | 1.90, 1.94 | 1 | 2*m |  |
| 37'' | 1.84, 1.91 | 1 | 2*m |  |
| 4' | 1.75, 1.77 | 1 | 2*m |  |
| 39' | 1.74, 1.72 | 1 | 2*m |  |
| 5' | 1.74, 1.66 | 1 | 2*m |  |
| 18'' | 1.68, 2.00 | 1 | 2*m |  |
| 42 | 1.64 | 3 | s |  |
| 17 | 1.63, 1.65 | 1 | 2*m |  |
| 34' | 1.61 | 1 | m |  |
| 16' | 1.53, 1.58 | 1 | 2*m |  |
| 12'' | 1.45, 1.51 | 1 | 2*m |  |
| 5'' | 1.42, 1.57 | 1 | 2*m |  |
| 4'' | 1.42, 1.44 | 1 | 2*m |  |
| 39'' | 1.42, 1.45 | 1 | 2*m |  |
| 33'' | 1.35 | 1 | m |  |
| 34'' | 1.05 | 1 | m |  |
| 16'' | 1.03, 1.30 | 1 | 2*m |  |
| 35 | 0.99, 0.92 | 3 | d, m | 6.4 |
| 38 | 0.96 | 3 | t | 7.5 |
| 30'' | 0.95, 0.93 | 1 | 2*m |  |
| 36 | 0.94, 0.88 | 3 | 2*m |  |
| 41 | 0.87, 0.91 | 3 | 2*m |  |
| 40 | 0.86 | 3 | m |  |

**Table S14**. ^13^C-NMR (150 MHz, CDCl_3_) spectrum annotation for 19-ethyl-19-desmethyl-FK520 (**7**).

19-ethyl-19-desmethyl-Fk520 (**7**)

| **Position** | **Chemical shift (ppm)** | **Number of carbons** | **Multiplicity** |
| --- | --- | --- | --- |
| 22 | 213.8, 213.1 | 1 | 2*s |
| 9 | 196.0, 193.7 | 1 | 2*s |
| 1 | 169.0, 168.9 | 1 | 2*s |
| 8 | 164.8, 165.6 | 1 | 2*s |
| 19 | 144.1, 144.8 | 1 | 2*s |
| 27 | 132.3, 131.6 | 1 | 2*s |
| 28 | 129.2, 129.8 | 1 | 2*s |
| 20 | 122.7, 123.1 | 1 | 2*s |
| 10 | 96.9, 98.6 | 1 | 2*s |
| 31 | 84.14, 84.17 | 1 | 2*s |
| 26 | 76.9, 78.0 | 1 | 2*s |
| CDCl_3_ | 77.0 |  | t |
| 15 | 75.2, 77.2 | 1 | 2*s |
| 13 | 73.74, 73.71 | 1 | 2*s |
| 32 | 73.53, 73,50 | 1 | 2*s |
| 14 | 72.8 | 1 | s |
| 24 | 70.2, 68.7 | 1 | 2*s |
| 44 | 56.9, 57.7 | 1 | 2*s |
| 2 | 56.63, 52.7 | 1 | 2*s |
| 45 | 56.61, 56.5 | 1 | 2*s |
| 43 | 56.3, 55.9 | 1 | 2*s |
| 21 | 54.3, 54.9 | 1 | 2*s |
| 18 | 45.3, 45.4 | 1 | 2*s |
| 23 | 42.8, 43,4 | 1 | 2*s |
| 25 | 39.7, 40.2 | 1 | 2*s |
| 6 | 39.3, 44.1 | 1 | 2*s |
| 30 | 34.86 | 1 | s |
| 29 | 34.84, 34.88 | 1 | 2*s |
| 11 | 34.5, 34.1 | 1 | 2*s |
| 16 | 32.8, 35.1 | 1 | 2*s |
| 12 | 32.7, 32.2 | 1 | 2*s |
| 33 | 31.2 | 1 | s |
| 34 | 30.63, 30.58 | 1 | 2*s |
| 3 | 27.5, 26,3 | 1 | 2*s |
| 17 | 26.4, 26.8 | 1 | 2*s |
| 5 | 24.55, 24.58 | 1 | 2*s |
| 39 | 24.55, 24.7 | 1 | 2*s |
| 37 | 22.0, 22.9 | 1 | 2*s |
| 4 | 21.2. 20.8 | 1 | 2*s |
| 36 | 20.7, 20.4 | 1 | 2*s |
| 35 | 16.2, 15.9 | 1 | 2*s |
| 42 | 14.3, 14.0 | 1 | 2*s |
| 38 | 12.7, 12.9 | 1 | 2*s |
| 40 | 11.8, 11.9 | 1 | 2*s |
| 41 | 9.5, 9.8 | 1 | 2*s |


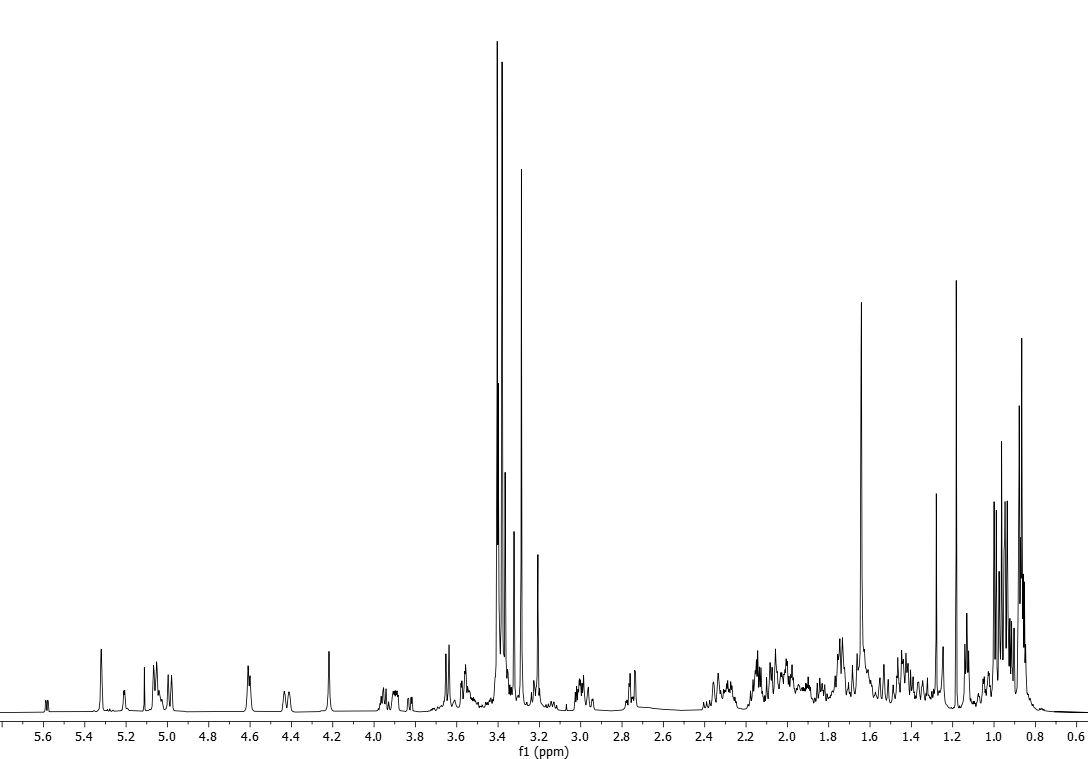

**Fig S26**. ^1^H-NMR (600 MHz, CDCl_3_) spectrum for 19-ethyl-19-desmethyl-FK520 (**7**).


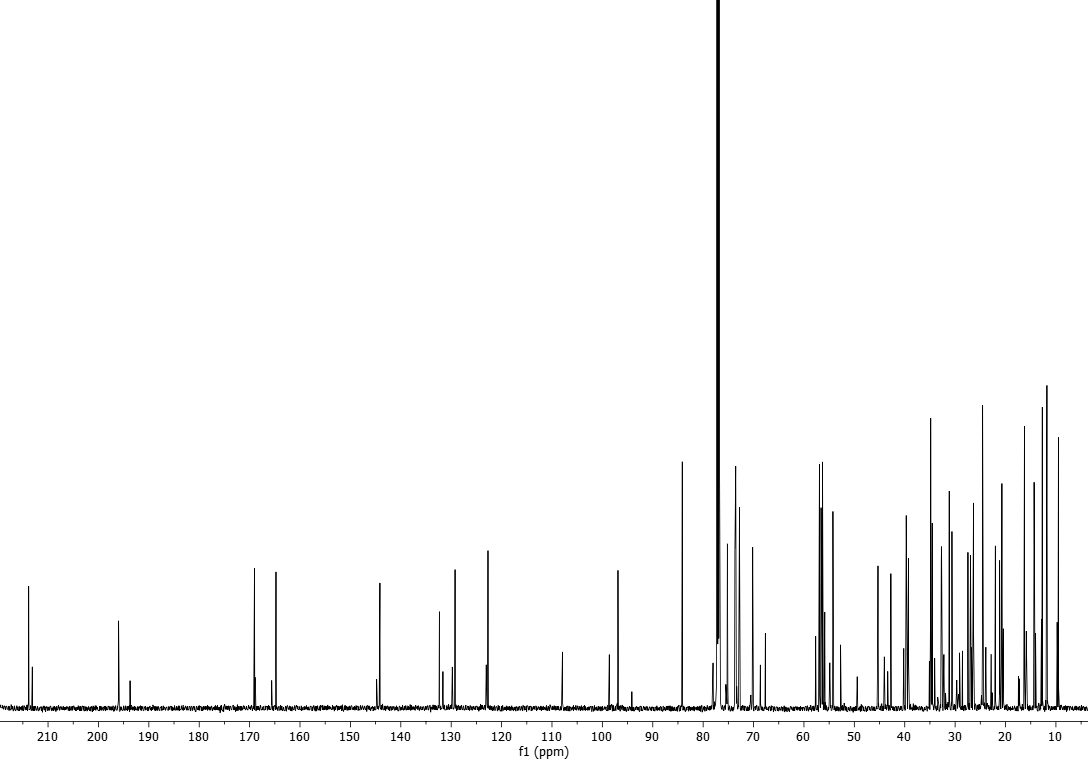


**Fig S27**. ^13^C-NMR (150 MHz, CDCl_3_) spectrum for 19-ethyl-19-desmethyl-FK520 (**7**).


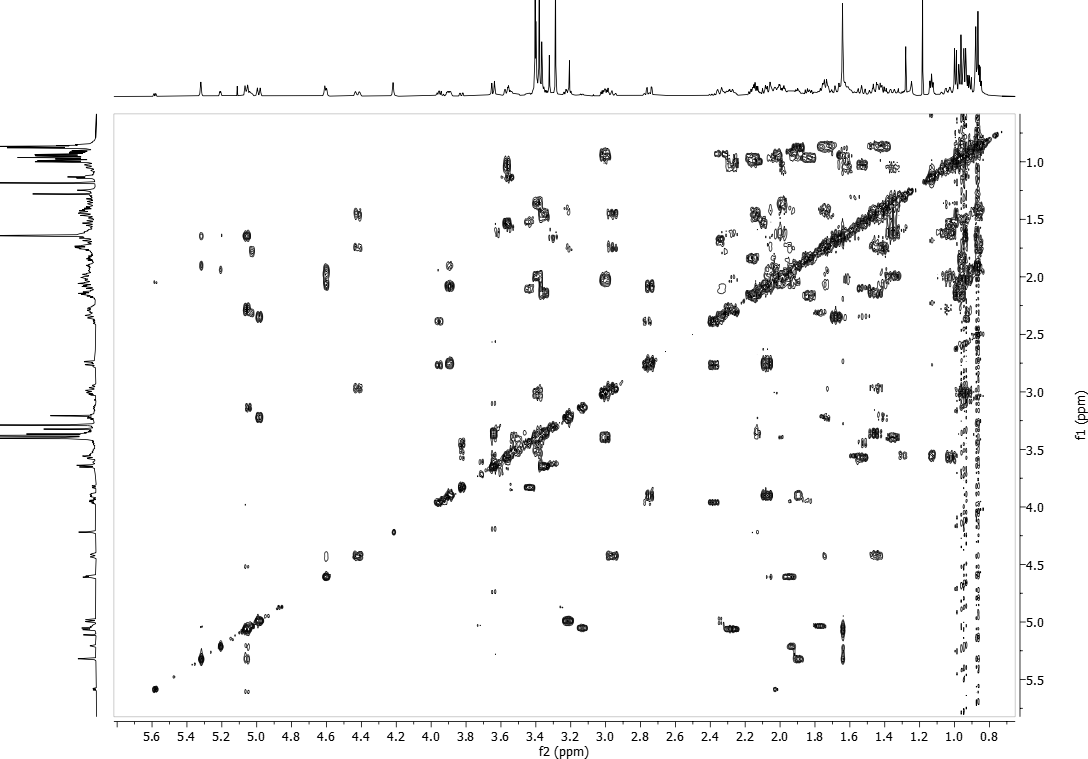


**Fig S28**. COSY 2D spectrum for 19-ethyl-19-desmethyl-FK520 (**7**).


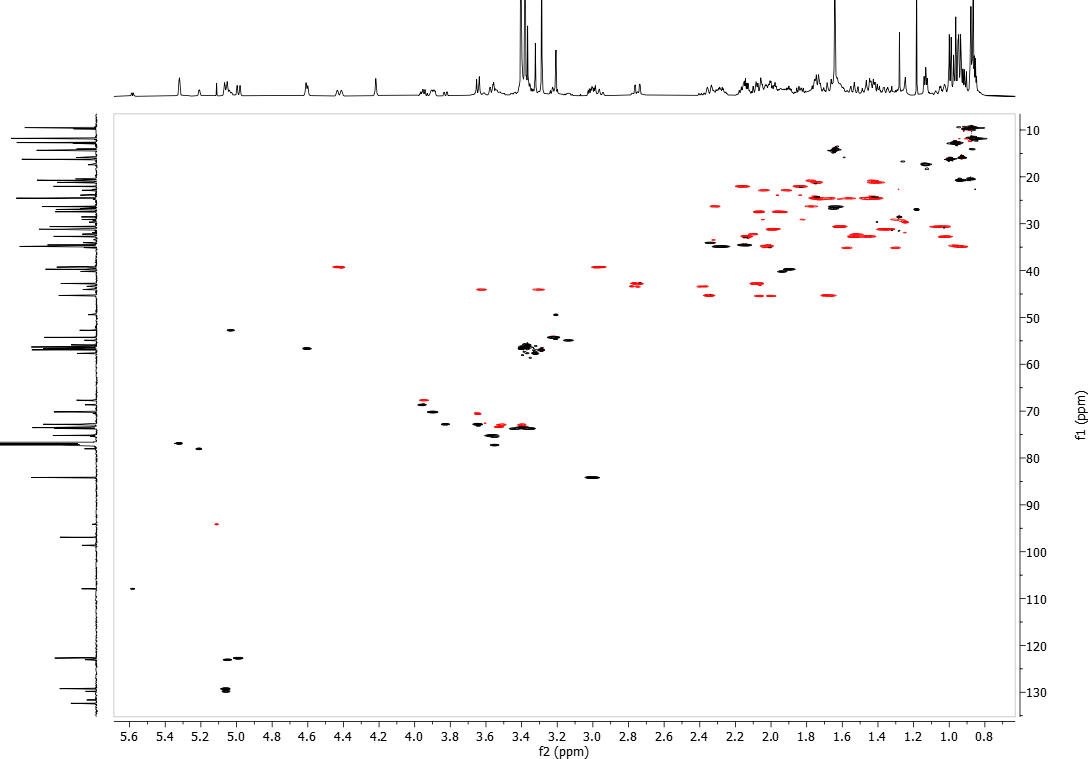


**Fig S29**. HSQC 2D spectrum for 19-ethyl-19-desmethyl-FK520 (**7**).


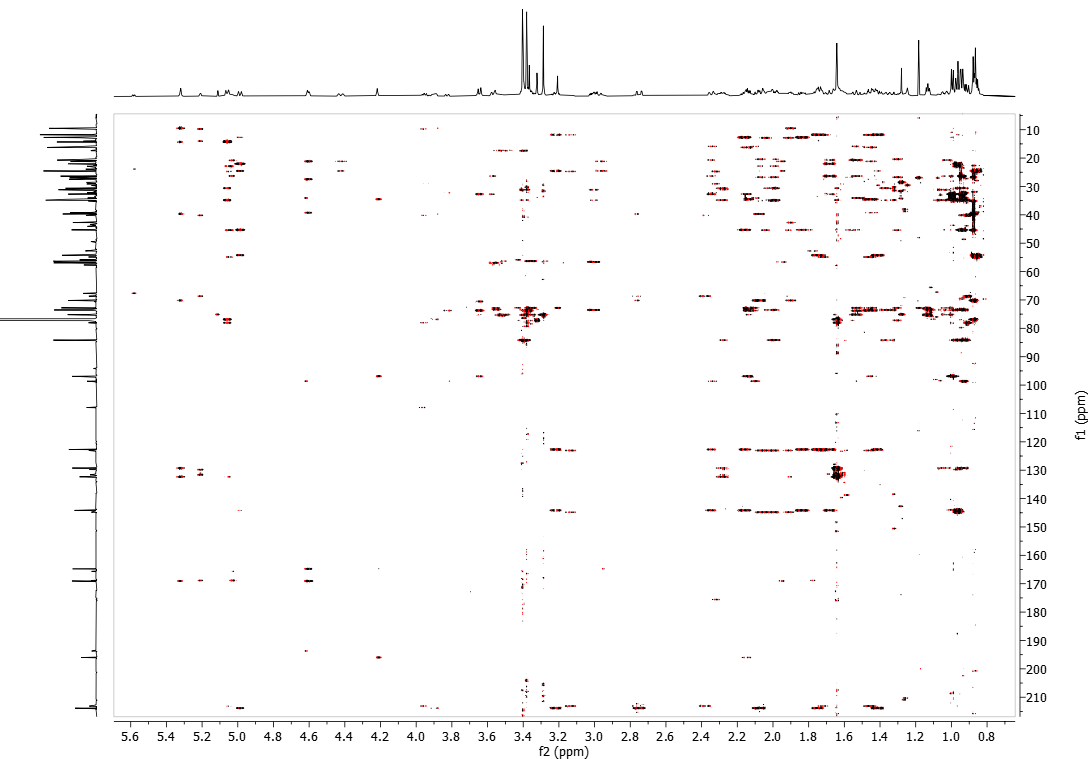


**Fig S30**. HMBC 2D spectrum for 19-ethyl-19-desmethyl-FK520 (**7**).
